# Efficacy and safety of tonic motor activation (TOMAC) for medication-refractory restless legs syndrome: a randomized clinical trial

**DOI:** 10.1093/sleep/zsad190

**Published:** 2023-07-17

**Authors:** Richard K Bogan, Asim Roy, Jerrold Kram, Joseph Ojile, Russell Rosenberg, J Douglas Hudson, H Samuel Scheuller, John W Winkelman, Jonathan D Charlesworth

**Affiliations:** Bogan Sleep Consultants, LLC, Columbia, SC, USA; Ohio Sleep Medicine Institute, Dublin, OH, USA; California Center for Sleep Disorders, San Leandro, CA, USA; Clayton Sleep Institute, LLC, St. Louis, MO, USA; NeuroTrials Research Inc., Atlanta, GA, USA; FutureSearch Trials of Neurology, Austin, TX, USA; Delta Waves, Inc., Colorado Springs, CO, USA; Massachusetts General Hospital, Boston, MA, USA; Noctrix Health, Inc., Pleasanton, CA, USA

**Keywords:** bioelectronic, neurological disorder, neuromodulation, peripheral nerve stimulation, restless legs syndrome, sleep disorder

## Abstract

**Study Objectives:**

The purpose of this study was to evaluate the efficacy and safety/tolerability of bilateral high-frequency tonic motor activation (TOMAC) in patients with medication-refractory restless legs syndrome (RLS).

**Methods:**

RESTFUL was a multicenter, randomized, double-blind, sham-controlled trial in adults with medication-refractory moderate-to-severe primary RLS. Participants were randomized 1:1 to active or sham TOMAC for a double-blind, 4-week stage 1 and all received active TOMAC during open-label, 4-week stage 2. The primary endpoint was the Clinical Global Impressions-Improvement (CGI-I) responder rate at the end of stage 1. Key secondary endpoints included change to International RLS Study Group (IRLS) total score from study entry to the end of stage 1.

**Results:**

A total of 133 participants were enrolled. CGI-I responder rate at the end of stage 1 was significantly greater for the active versus sham group (45% vs. 16%; Difference = 28%; 95% CI 14% to 43%; *p* = .00011). At the end of stage 2, CGI-I responder rate further increased to 61% for the active group. IRLS change at the end of stage 1 improved for the active versus sham group (−7.2 vs. −3.8; difference = −3.4; 95% CI −1.4 to −5.4; *p* = .00093). There were no severe or serious device-related adverse events (AEs). The most common AEs were mild discomfort and mild administration site irritation which resolved rapidly and reduced in prevalence over time.

**Conclusions:**

TOMAC was safe, well tolerated, and reduced symptoms of RLS in medication-refractory patients. TOMAC is a promising new treatment for this population.

**Clinical Trial:**

Noninvasive Peripheral Nerve Stimulation for Medication-Refractory Primary RLS (The RESTFUL Study); clinicaltrials.gov/ct2/show/NCT04874155; Registered at ClinicalTrials.gov with the identifier number NCT04874155.

## Graphical Abstract

**Graphical Abstract:**
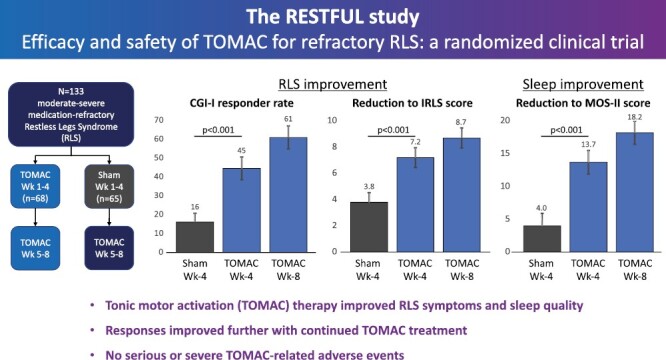

**Statement of Significance**

Tonic motor activation (TOMAC) is a novel wearable therapeutic device that reduces restless legs syndrome (RLS) symptoms by electrically stimulating specific fibers of the peroneal nerve to activate the tibialis anterior muscle. Medication-refractory RLS requires novel effective and safe treatment options to improve RLS symptoms, sleep, and quality of life. This article presents results from the first randomized, double-blind, sham-controlled trial evaluating the efficacy and safety of TOMAC in adults with medication-refractory RLS. TOMAC treatment produced clinically meaningful reductions in RLS symptoms and had excellent safety and tolerability. Response to TOMAC improved over time, motivating longer-duration open-label studies in the future. These results demonstrate for the first time that TOMAC is a safe and efficacious treatment for refractory RLS.

## Introduction

Restless legs syndrome (RLS) is a neurological condition and sleep disorder characterized by a distressing urge to move the legs that increases during immobility and worsens during the night [1]. Nocturnal RLS symptoms result in sleep disturbances that cause sleep deprivation [2] and thereby increase the risks of associated chronic health conditions including dementia, hypertension, heart failure, and diabetes mellitus [3, 4]. Psychological consequences of RLS include depressed mood, increased risk of suicide and self-harm, difficulty learning, and lack of motivation [5, 6]. An estimated 2%–3% of adults in the United States (US) and Europe suffer from moderate-to-severe RLS [7], in which symptoms present with sufficient frequency and severity to significantly reduce quality of life [8].

There is a large and growing population of patients with medication-refractory RLS, defined as RLS that is unresponsive to monotherapy with tolerable doses of first-line agents due to reduction in efficacy, augmentation, or adverse effects [9]. Long-term use of the most common first-line RLS medications, dopamine agonists (DAs), frequently leads to augmentation, defined as paradoxical increases in RLS symptoms relative to natural progression of the disorder [10]. Up-titration of DAs can provide transient relief, but exacerbates augmentation and increases the risk of impulse control disorders including compulsive gambling and hypersexuality [9]. Other commonly prescribed first-line RLS medications, alpha-2-delta ligands, have side effects that limit tolerability [9].

Till date, no treatments have been approved by the US Food and Drug Administration (FDA) for patients who are refractory to RLS medication. Consensus expert recommendations suggest that opioids can be effective under careful supervision by a specialist, but side effects can limit tolerability [11, 12]. Moreover, the ongoing opioid epidemic has contributed to hesitancy in prescribing off-label opioids. As a result, a large population of refractory RLS patients continue to suffer with severe RLS symptoms and sleep deprivation.

**Figure 1.**
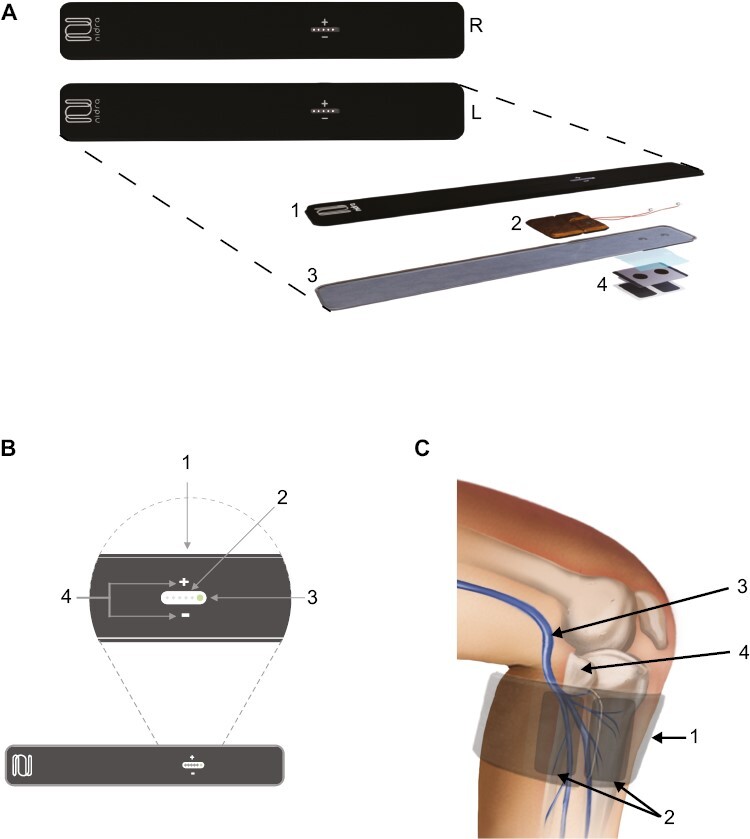
Investigational TOMAC system. (A) The investigational TOMAC system consists of two therapy units corresponding to the Right (R) and Left (L) legs. The exploded view illustrates the components of each therapy unit, consisting of the (1) compressive conduction garment outer side, (2) HF-TOMAC module, which includes the programmable pulse generator, user controls, and rechargeable battery, (3) compressive conduction garment leg-facing side, and (4) charge dispersing interface, which conducts electricity from the HF-TOMAC module to the leg and is shown in an exploded view including disposable protective liners on both sides. (B) The compressive conduction garment of each therapy unit includes the following components (1) Charge port for the rechargeable battery, (2) Intensity lights indicating the stimulation level relative to the titrated value, (3) Status light indicating stimulation and charging status, and (4) Control buttons used to turn therapy on and off and to adjust the stimulation level relative to the titrated value. (C) Depiction of (1) TOMAC therapy unit, shown in light gray, worn so that the (2) charge dispersing interfaces, shown in dark gray, are positioned over the (3) peroneal nerve near (4) the head of the fibula bone.

The TOMAC system (Figure 1; Noctrix Health, Pleasanton, CA, USA) is a novel nonpharmacological, noninvasive treatment consisting of a pair of therapy units that are worn externally and bilaterally over the peroneal nerve at the head of the fibula bone in the lower leg. TOMAC stimulates afferent peroneal nerve fibers in a comfortable and nondistracting pattern that evokes sustained increases in tibialis anterior muscle tone [13]. Through this established mechanism, TOMAC engages similar neuromuscular circuitry as voluntary leg movements such as walking or standing, which are known to suppress RLS symptoms, while remaining compatible with sleep [13, 14]. An earlier prototype of the TOMAC system showed promising results in a single-blind proof-of-concept study with a mixed population of 12 medication-naïve and 20 medication-refractory RLS patients [15]; in that study, 30-minute TOMAC stimulation sessions resulted in relief of RLS symptoms that began during stimulation and persisted after stimulation [15]. The purpose of the RESTFUL study, a multicenter, double-blind, sham-controlled, randomized trial, was to assess the efficacy and safety/tolerability of tonic motor activation (TOMAC) in medication-refractory RLS patients.

## Methods

### Study design

The RESTFUL study was a multicenter, prospective, randomized, double-blind, sham-controlled pivotal trial conducted at 7 sleep clinics in the United States. During stage 1 (Weeks 1–4), participants were randomized 1:1 to active or sham TOMAC treatment. The primary endpoint was assessed at the end of Stage 1. During stage 2 (Weeks 5–8), all participants were assigned to open-label active TOMAC treatment. There was no drug washout; participants were required to maintain a stable dose and schedule of RLS medication for the 30 days prior to study entry and throughout the duration of the study. Upon completion of the RESTFUL study, participants were invited to participate in a separate extension study protocol, the results of which are published separately [16]. The RESTFUL study was conducted in accordance with the International Conference on Harmonization guidelines on good clinical practice and the Declaration of Helsinki and was designed with input from the US FDA. A blinded independent medical monitor was responsible for adjudicating adverse events (AEs).

### Participants

Eligible participants were adults aged 22–79 years with medication-refractory moderate-to-severe primary RLS. Diagnosis was confirmed based on International RLS Study Group diagnostic criteria [14] and severity was determined by International RLS Study Group Rating Scale (IRLS) total score ≥15. Medication-refractory RLS was defined as having failed one or more medications commonly prescribed to treat RLS (ropinirole, pramipexole, gabapentin, pregabalin, gabapentin enacarbil, and/or rotigotine) for at least one of the following reasons: intolerable adverse effects, symptoms of augmentation, up-titration needed due to reduced efficacy, and insufficient response at maximum approved, recommended, or tolerated dosage. Additional key inclusion criteria were symptoms ≥2 nights per week, symptoms most significant in the lower legs and/or feet, and symptoms most significant at bedtime, after bedtime, and/or in the 2 hours before bedtime. Key exclusion criteria were unstable doses of RLS medications, sleep medications, or antidepressants, inadequately treated primary sleep disorders other than RLS, severe peripheral neuropathy affecting the lower legs, skin conditions affecting the application site, known allergy to device materials, active medical device implants, epilepsy, dialysis, and iron-deficient anemia, and prior experience with the study device or with any neurostimulation device to treat RLS. The protocol was amended on June 15, 2021 to allow participants with knee replacement implants (e.g. total knee arthroscopy) to participate in the study based on information that other forms of local electrical stimulation were well-tolerated by these patients [17, 18].

### Randomization and masking

Randomization was based on computer-generated permuted blocks of randomized lengths of 4 or 6, stratified by study center. A third-party statistician (Biostatistical Consulting Inc., Lexington, MA, USA), independent of all other study activities, generated the randomization sequence. Information about treatment assignment was concealed from participants, investigators, and research staff; no emergencies occurred that required breaking the blind. TOMAC systems were preprogrammed to active or sham mode prior to shipment to sites by a manufacturer that was not involved with any aspect of study conduct. All procedures and instructions were identical for active and sham treatment. Active and sham devices were physically identical and provided identical visual feedback during operation. A previously validated transient sham design was implemented [19]; both active and sham modes increased to the same intensity level in the first 20 s of each therapy session; afterwards, active mode remained active whereas sham mode reduced to zero within 10 s. Between stages 1 and 2, all devices were electronically configured to active mode by the research staff without revealing the previous mode to the participant or research staff. After stage 1, participants were asked to state their belief regarding treatment group assignment and if their belief was primarily due to perceived efficacy, side effects, or other factors.

### Procedures

The TOMAC system was comprised of two therapy units worn bilaterally on the lower legs (Figure 1). The system was designed to modulate afferent fibers of the peroneal nerve and evoke tonic motor activation of the tibialis anterior muscle, thereby mimicking the neural signals associated with voluntary leg movements. Each therapy unit included a high-frequency TOMAC pulse generator, electronic data logger, rechargeable battery, user controls, status lights, compressive conduction garment, and conductive charge-dispersing interface (Figure 1). Each conductive charge-dispersing interface included two electrodes, each with surface area 3.3 cm × 5.1 cm, which contained layers of charge-dispersing silver-coated film and adhesive hydrogel. Participants were trained to position therapy units so that the charge-dispersing interfaces covered the peroneal nerve at the head of the fibula bone, to self-administer a full 30-minute therapy session whenever they experienced distressing RLS symptoms, while prioritizing usage at bedtime, after bedtime, or in the 2 h before bedtime, and to use a maximum of 4 sessions (120 min) per day; training lasted approximately 20 min. At study entry, trained clinic staff completed a titration procedure for each leg that involved cycling the stimulation intensity to determine the maximal stimulation intensity that was subjectively compatible with sleep; this titrated intensity level was then programmed into each device for in-home use. The duration of each stimulation session was set to 30 min, after which stimulation automatically shut off.

### Outcomes

Efficacy outcome measures were centrally assessed for the intent-to-treat (ITT) population, which comprised all eligible subjects who passed screening and were randomized. The primary endpoint was the Clinical Global Impressions-Improvement (CGI-I) responder rate at Week 4 (end of stage 1), with responders defined as having been rated by the clinician as “much improved” or “very much improved.” The CGI-I was administered by investigators experienced in the diagnosis and treatment of RLS. Prespecified key secondary endpoints, all completed by the participant and assessed at Week 4 relative to study entry, were ordered as follows: Patient Global Impressions-Improvement (PGI-I) responder rate, change in total IRLS score, change in Medical Outcomes Study Sleep Problem Indices II (MOS-II) and I (MOS-I) scores, mean CGI-I score, and change in IRLS Question #7 score (frequency of RLS symptoms). Change in IRLS question #7 score from baseline to Week 8 was added as a key secondary endpoint before breaking the blind. Descriptive analyses of CGI-I, PGI-I, IRLS, MOS-I, and MOS-II at Week 8 were also prespecified.

Adherence to device usage was assessed automatically by the device. Patient satisfaction with treatment was assessed using several questions with yes/no response options.

Safety outcome measures were centrally assessed. The count and proportion of participants reporting AEs with new onset or worsening (relative to baseline) were compared between treatment groups and stages. AEs were coded and summarized at the participant level by Medical Dictionary for Regulatory Activities System Organ Class and Preferred Terms and by seriousness, severity, and relationship to the device.

Participants self-identified their race and ethnicity on a survey.

### Statistical analysis

The planned enrollment—prespecified in the protocol—was 132 participants (66 per treatment group); this was intended to yield 112 subjects with completed data assuming a 15% drop-out rate. The sample size was chosen to provide 85% power to detect a statistically significant difference between treatment groups at a one-sided alpha level of 0.025, based on effect sizes for CGI-I and IRLS from a previous proof-of-concept study [15]. The only specified early stopping rule was in the case of an unanticipated serious adverse device effect; no such event occurred in the study. Therefore, enrollment for the study concluded on the date that the enrollment target was met (February 10, 2022); two participants at separate centers were enrolled on that date and thus the total enrollment was 133 instead of 132. The trial ended when all enrolled participants completed their participation in the study.

Efficacy analyses were performed on the ITT population. Multiple imputation was used for the primary endpoint and no imputation was used for the secondary endpoints. Hierarchical testing was employed to account for multiplicity. CGI-I and PGI-I responder rates were compared using a one-sided normal approximation test for comparison of two proportions; mean scores on the IRLS, MOS-II, MOS-I, CGI-I, and IRLS question 7 were compared using a one-sided, two-sample *t*-test. A one-sided alpha level of 0.025 defined statistical significance for all comparisons.

The safety analysis population included all participants who received any dose of study treatment, including participants who underwent titration but failed screening. Participants were analyzed based on actual treatment received. Rates of AEs were compared using one-sided, two-sample *t*-tests with no accounting for multiplicity. Statistical analyses were conducted using SAS version 9.4 (Cary, NC, USA).

The study protocol and statistical analysis plan are available in Supplementary Material 1 and Supplementary Material 2, respectively.

### Exploratory analysis

Exploratory analyses were conducted to determine which components of sleep were improved by active versus sham TOMAC at the end of stage 1. Post-hoc *t*-tests were conducted for each MOS item included in the MOS-I or MOS-II (Items 1, 3, 4, 5, 6, 7, 8, 9, 12). For each item, a one-sided *t*-test with alpha level of 0.025 was conducted and the Holm–Bonferroni method was used to account for multiple comparisons.

For comparing RLS improvement depending on baseline RLS severity, baseline IRLS scores of 11–20 were considered moderate, 21–30 severe, and 31–40 very severe. Since a minimum IRLS score of 15 was required for study entry, 15–20 was the actual range of moderate baseline symptoms.

Participants were asked to rate the duration of RLS symptom relief—if any—that persisted after completion of each 30-minute TOMAC session. Participants were asked to rate this only for TOMAC sessions administered more than 2 h before bedtime, due to the challenges with accurately estimating duration of symptom relief during the sleep period. Multiple choice options ranged from “no relief” after TOMAC to “>2 hours of relief” after TOMAC.

Objective exposure to therapy was assessed through analysis of therapy unit device logs which recorded the timing, duration (minutes), and participant-selected intensity (current, mA) for each therapy session. Since participants were instructed to use the device only with RLS symptoms, objective adherence was assessed by comparing daily frequency of completed sessions to daily frequency of RLS symptoms based on responses to IRLS question #7.

### Standard protocol approvals, registrations, and patient consents

The study protocol and informed consent were approved by a central institutional review board (Advarra, Columbia, MD, USA). All participants provided informed consent. The trial was preregistered (ClinicalTrials.gov number, NCT04874155) on May 5, 2021.

### Data availability

The deidentified data that support the findings of this study are available from the corresponding author upon reasonable request.

## Results

### Participant characteristics

**Figure 2.**
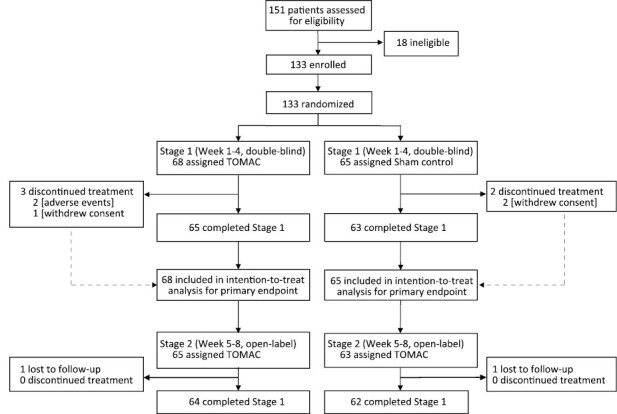
Enrollment, randomization, and treatment.

Between May 6, 2021, and February 10, 2022, 151 individuals were screened for eligibility, 18 were deemed ineligible, and the remaining 133 were enrolled (Figure 2). Of the 133 enrolled participants, 68 were assigned to active TOMAC for stage 1 and the remaining 65 were assigned to sham. All 133 participants were included in the ITT population for assessment of the primary endpoint and all received the intended treatment. During stage 1, 5 participants discontinued treatment including 3 in the active arm (2 due to AEs and 1 withdrew consent) and 2 in the sham arm (2 withdrew consent). The remaining 128 participants (96%) entered open-label stage 2. In stage 2, no participant discontinued treatment, 2 participants were lost to follow-up, and the remaining 126 participants (98%) completed the study. Complete data for the primary and key secondary efficacy endpoints was available for all participants who completed each stage (128 for stage 1, 126 for stage 2).

Table 1 summarizes demographics and disease characteristics. Mean age of participants was 57.5 years (SD 11.4) and 60% of participants were female. Mean duration of RLS was 22.6 years (SD 14.9). Mean IRLS total score at baseline was 25.2 (SD 5.3) for participants allocated to TOMAC and 25.4 (SD: 5.3) for participants allocated to sham. Participants had taken RLS medication for a mean duration of 11.4 years (SD 8.2) and were refractory to an average of 1.77 medications; 86% (*n* = 115) were refractory to DAs, 57% (*n* = 76) were refractory to alpha-2-delta ligands, and 44% (*n* = 58) were refractory to both classes. Of the 120 participants (90%) with moderate-to-severe RLS despite taking RLS medication at study entry, 115 (96%) maintained a stable medication regimen throughout the study, 2 took a different dosage on 1 day, 1 took a different dosage on 4 days, and 2 changed medication during the study (both in stage 2).

**Table 1.** Demographic and clinical characteristics

Variable	TOMAC (*n* = 68)	Sham (*n* = 65)	All (*n* = 133)^a^
Sex, No. (%)			
Female	39 (57)	41 (63)	80 (60)
Male	29 (43)	24 (37)	53 (40)
Age, mean (SD), y	56.3 (11.0)	58.6 (11.8)	57.5 (11.4)
Age ≥65 years, No. (%)	17 (25%)	23 (35%)	40 (30%)
Race, No. (%)			
American Indian or Alaska Native	0	0	0
Asian	2 (3)	0	2 (2)
Black or African American	2 (3)	0	2 (2)
Native Hawaiian or Pacific Islander	0	0	0
White	64 (94)	65 (100)	129 (97)
Ethnicity, No. (%)			
Hispanic or Latino	4 (6%)	4 (6%)	8 (6%)
Not Hispanic or Latino	64 (94%)	61 (94%)	125 (94%)
Duration of RLS symptoms, mean (SD), y	22.5 (14.4)	22.8 (15.6)	22.6 (14.9)
IRLS total score, mean (SD)	25.2 (5.3)	25.4 (5.3)	25.3 (5.3)
Classes of refractory RLS medications, No. (%)			
Dopamine agonists	61 (90%)	54 (83%)	115 (86%)
Alpha-2-delta ligands	36 (53%)	40 (62%)	76 (57%)
Both	29 (43%)	29 (45%)	58 (44%)
Classes of current RLS medication, No. (%)			
Dopamine agonists	47 (69%)	48 (74%)	95 (71%)
Alpha-2-delta ligands	18 (26%)	16 (25%)	34 (26%)
Opioids	4 (6%)	7 (11%)	11 (8%)
Benzodiazepines	1 (1%)	1 (2%)	1 (2%)
Other	7 (10%)	7 (11%)	14 (11%)
No prescribed RLS medications	8 (12%)	5 (8%)	13 (10%)

Abbreviations: IRLS, International RLS Study Group Rating Scale; RLS, restless legs syndrome; SD, standard deviation; TOMAC, tonic motor activation.

### Primary and key secondary efficacy endpoints

**Figure 3.**
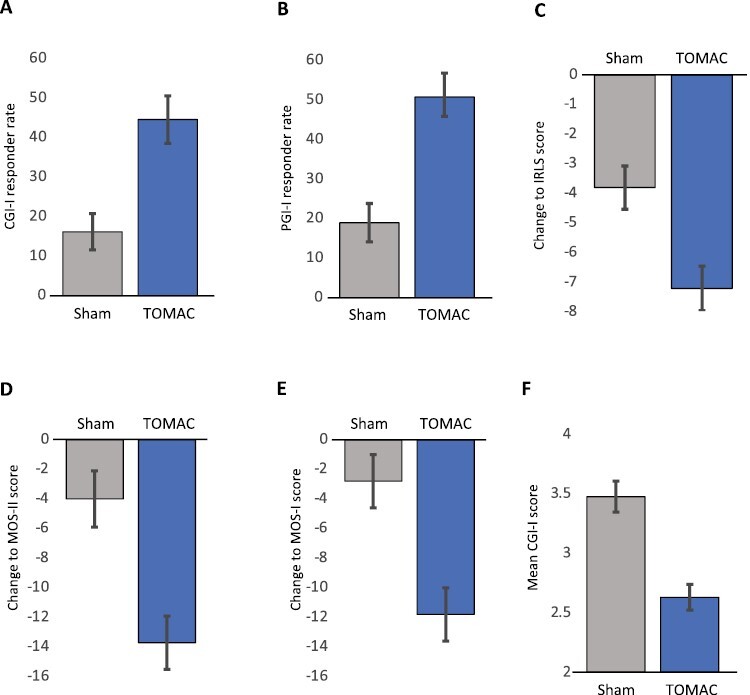
Comparison of Efficacy Endpoints between TOMAC and Sham. Comparison of response to TOMAC and Sham assessed at Week 4 compared to baseline for the following efficacy endpoints: A. CGI-I responder rate, B. PGI-I responder rate, C. IRLS total score, D. MOS-II score, E. MOS-I score, F. CGI-I mean score. Error bars represent standard error of means and proportions.

Results of primary and key secondary endpoints are presented in Table 2. The primary endpoint was met; for clinician-rated CGI-I responder rate assessed at the end of stage 1, 45% of participants assigned to TOMAC were responders compared to 16% for sham control (Figure 3A, Difference = 28%; 95% CI 14% to 43%; *p* = .00011). Each secondary endpoint was also met. PGI-I responder rate was 51% for TOMAC compared to 19% for sham (Figure 3B, Difference = 32%; 95% CI 17% to 47%; *p* < .0001). Mean IRLS total score change was −7.2 points for TOMAC compared to −3.8 points for sham (Figure 3C, Difference = −3.4; 95% CI −1.4 to −5.4; *p* = .00093), demonstrating a reduction in RLS severity with TOMAC. Mean MOS-II change was −13.7 for TOMAC compared to −4.0 for sham (Figure 3D, Difference = −9.7; 95% CI −4.7 to −14.7; *p* = .00017) and mean MOS-I change was −11.8 for TOMAC compared to −2.8 for sham (Figure 3E, Difference = −9.1; 95% CI −4.1 to −14.1; *p* = .00036), demonstrating a reduction in sleep problems with TOMAC. Mean CGI-I score at Week 4 was 2.6 for TOMAC compared to 3.5 for sham (Figure 3F, Difference = 0.85; 95% CI 0.52 to 1.179; *p* < .0001). The score on IRLS question #7 reduced from 3.6 at baseline to 2.7 at Week 8 for participants assigned to TOMAC (Difference = 0.89; 95% CI 0.63 to 1.15; *p* < .0001), demonstrating a reduction in frequency of RLS symptoms.

**Table 2.** Efficacy outcome measures by treatment group

Outcome	Stage 1 Treatment Assignment		Between Groups Difference (95% CI)	P value
	TOMAC (*n* = 68)	Sham (*n* = 65)		
**Primary Endpoint**				
CGI-I responder rate at Week 4, No. (%)				
Observed	29 (45%)	10 (16%)	NA	NA
Imputed	45%	16%	28% (14% to 43%)	.00011
**Key Secondary Endpoints**				
PGI-I responder rate at Week 4, No. (%)`	33 (51%)	12 (19%)	32% (17% to 47%)	<.0001
IRLS total score change from baseline to Week 4, mean (SD)	−7.2 (6.2)	−3.8 (5.9)	−3·4 (−1.4 to −5.4)	.00093
MOS-II score change from baseline to Week 4, mean (SD)	−13.7 (14·9)	−4.0 (14.8)	−9·7 (−4.7 to −14.7)	.00017
MOS-I score change from baseline to Week 4, mean (SD)	−11.8 (14·7)	−2.8 (14.9)	−9.1 (−4.1 to −14.1	.00036
CGI-I mean (SD) at Week 4, assessed relative to baseline	2.6 (1.1)	3.5 (0.9)	0.85 (0.52 to 1.18)	<.0001
Change in IRLS question #7 score from baseline to Week 8, mean (SD)	−0.9 (1.1)	−0.6 (1.2)	0.89 (0.63 to 1.15)	<.0001
**Endpoints Assessed at End of Open-label Stage**				
CGI-I at Week 8, No. (%)				
Responder rate (at least Much Improved)	39 (61%)	39 (64%)	NA	NA
Improvement rate (at least Minimally Improved)	57 (89%)	54 (89%)	NA	NA
PGI-I at Week 8, No. (%)				
Responder rate (at least Much Improved)	39 (61%)	37 (61%)	NA	NA
Improvement rate (at least Minimally Improved)	58 (91%)	49 (80%)	NA	NA
IRLS total score change from baseline to Week 8, mean (SD)	−8.7 (6.3)	−7·0 (6.3)	NA	NA
MOS-II score change from baseline to Week 8, mean (SD)	−18.2 (14·5)	−13·2 (14.7)	NA	NA
MOS-I score change from baseline to Week 8, mean (SD)	−15.7 (14·5)	−11·5 (14.8)	NA	NA
CGI-I score at Week 8 relative to baseline, mean (SD)	2.2 (1.0)	2.3 (1.0)	NA	NA

Abbreviations: CGI-I, Clinical Global Impressions-Improvement; CI, confidence interval; IRLS, International RLS Study Group Rating Scale; MOS-I, Medical Outcomes Study Sleep Problem Index I; MOS-I, Medical Outcomes Study Sleep Problem Index II; NA, not applicable; PGI-I, Patient-Global Impressions-Improvement; TOMAC, tonic motor activation.

### Efficacy outcomes at end of open-label treatment

Additional prespecified analyses were performed to assess responses for each efficacy outcome measure at the end of open-label stage 2 at Week 8 (Table 2). For participants assigned to TOMAC in stage 1, each outcome measure showed further improvement during stage 2 as they continued to use TOMAC; CGI-I responder rate increased from 45% to 61%, PGI-I responder rate increased from 51% to 61%, IRLS change reduced from −7.2 to −8.7 points, MOS-II change reduced from −13.7 to −18.2 points, and MOS-I change reduced from −11.8 to −15.7 points. For participants assigned to sham in stage 1, each outcome measure showed large improvements in stage 2 as the participants switched from sham to TOMAC; CGI-I responder rate increased from 16% to 64%, PGI-I responder rate increased from 19% to 61%, IRLS change reduced from −3.8 to −7.0 points, MOS-II change reduced from −4.0 to −13.2 points, and MOS-I change reduced from −2.8 to −11.5 points. At the end of stage 2, 89% of participants showed improvement (score of 3 or lower) on the CGI-I and 86% reported improvement on the PGI-I. The percentage of participants with IRLS reduction of 3 points or greater was 75% at Week 4 and 88% at Week 8 of TOMAC therapy and the interquartile range for IRLS change was −3.0 to −11.0 at Week 4 and −4.8 to −11.3 at Week 8.

### Safety and tolerability

AEs were typically mild and resolved rapidly without medical intervention (Table 3). There were no serious or severe device-related AEs. Only two categories of AEs occurred in greater than 5% of participants, both of which were mild (Grade 1 severity): discomfort (28% for active TOMAC stage 1, 8% for sham stage 1, and 11% for stage 2) and administration site irritation (9% for active TOMAC stage 1, 8% for sham stage 1, and 2% for stage 2). Two participants in active TOMAC stage 1 (3%) and zero participants in stage 2 discontinued treatment due to an AE; one due to a foot fracture secondary to blunt force trauma (categorized as not device-related) and the other due to knee pain and swelling (categorized as possibly device-related). Of the 89 AEs categorized as device-related, 99% (88) were Mild (Grade 1), 1% (1) was Moderate (Grade 2), none were Severe or Serious, and 72% resolved within 1 day or less. Device-related AEs resolved with minimal intervention; the most common actions taken were adjustment of stimulation intensity and adjustment of therapy unit positioning. For participants assigned to active TOMAC, AE rates decreased from stage 1 to stage 2; specifically, device-related AEs reduced from 38% to 9% and discomfort decreased from 28% to 4%.

**Table 3.** Treatment-emergent adverse events by group and stage

Variable	Stage 1		Stage 2 (grouped by Stage 1 assignment)		
	TOMAC (*n* = 68)	Sham (*n* = 65)	TOMAC (*n* = 68)	Sham (*n* = 65)	All (*n* = 133)
AE					
Any	32 (47%)	18 (28%)	12 (18%)	22 (34%)	34 (26%)
Device-related	26 (38%)	14 (22%)	6 (9%)	15 (23%)	21 (16%)
Serious AE					
Any	1 (1%)	0	0	1 (1%)	1 (1%)
Device-related	0	0	0	0	0
Discontinuation due to AE					
Any	2 (3%)	0	0	0	0
Device-related	1 (1%)	0	0	0	0
MedDRA Preferred Term^a^					
Discomfort	19 (28%)	5 (8%)	3 (4%)	11 (17%)	14 (11%)
Administration site irritation	6 (9%)	5 (8%)	0	2 (3%)	2 (2%)
AE severity					
Grade 1	31 (46%)	18 (28%)	10 (15%)	18 (28%)	28 (21%)
Grade 2	2 (3%)	0	2 (3%)	3 (5%)	5 (4%)
Grade 3	1 (1%)	0	0	1 (2%)	1 (1%)
Grade 4 or 5	0	0	0	0	0

^a^MedDRA preferred terms occurring in 5% of more of participants are shown.

Abbreviations: AE, adverse event; MedDRA, Medical Dictionary for Regulatory Activities; TOMAC, tonic motor activation.

Blinding was assessed at the end of stage 1. For subjects assigned to active TOMAC, 35 (54%) guessed treatment, 9 (14%) guessed sham, and 21 (32%) responded “don’t know.” For subjects assigned to sham, 10 (16%) guessed treatment, 40 (64%) guessed sham, and 13 (21%) responded “don’t know.” Guesses of treatment or sham (*n* = 94, 73%) were predominantly explained by perceived efficacy (*n* = 39, 41%) or lack of efficacy (*n* = 41, 44%), as opposed to side effects (*n* = 1, 1%) or other factors (*n* = 11, 12%). Therefore, the robust potency of treatment made it challenging to maintain blinding but other factors did not have a significant impact on blinding.

### Exposure to treatment

Objective measurement of TOMAC usage indicated high adherence with instructions to use devices on all days with RLS symptoms. In stage 1, participants assigned to TOMAC completed one or more device sessions on 101% of days with RLS symptoms (71% of total days). In stage 2, participants completed one or more device sessions on 97% of days with RLS symptoms (64% of total days).

For subjects assigned to TOMAC, stimulation intensity was stable over time with no evidence of habituation. Average participant-selected stimulation intensity was 29.9 mA (SD: 7.0 mA) at Week 1 and 30.7 (SD: 6.9 mA) at Week 8 and there was minimal week-to-week variation within participants (week-to-week SD: 1.1 mA). Stability of average weekly intensity could not be attributed to inability or unwillingness to adjust intensity; 98% of participants used multiple intensity levels at some point during the trial.

### Exploratory analyses

To determine which specific components of sleep were improved in response to TOMAC treatment, we conducted exploratory post hoc analysis on each of the items included in the MOS-I and MOS-II at the end of stage 1, using the Holm–Bonferroni method to account for multiple comparisons. TOMAC resulted in statistically significant reductions in MOS item 7 (trouble falling asleep) and MOS item 9 (trouble staying awake during the day) relative to sham control. Change to MOS item 7 was −20.6 points for TOMAC compared to −2.9 points for sham (Difference = −17.8; 95% CI −8.5 to −27.1, *p* = .0018) and change to MOS item 9 was −11.7 points for TOMAC compared to −0.6 points for Sham (Difference = −11.1; CI −3.6 to −18.5, *p* = .022). These results indicate that TOMAC reduced the RLS symptoms of trouble falling asleep and daytime sleepiness.

We compared RLS improvement at Week 8 for participants with moderate, severe, or very severe baseline symptoms, as defined by IRLS score at study entry. All three subgroups showed substantial improvement. Mean IRLS change at Week 8 was −4.2 points for participants with moderate baseline symptoms (*n* = 28), −8.8 for severe (*n* = 83), and −9.1 for very severe. Mean IRLS percent reduction was −23% for moderate, −34% for severe, and −28% for very severe. CGI-I responder rate was 63% for moderate, 56% for severe, and 86% for very severe.

Responders were asked to quantify the duration of RLS symptom reduction after each individual 30-minute TOMAC session. Due to the difficulty of quantifying symptoms while asleep, participants were specifically asked to quantify duration of relief for TOMAC sessions administered more than 2 hours before bedtime. Among CGI-I responders (CGI-I of 1 or 2 at Week 8) who used the device in this context (*n* = 36), 67% indicated that symptoms were reduced for >2 hours after TOMAC sessions and 86% indicated that symptoms were reduced for >30 minutes after TOMAC sessions.

Satisfaction was high, with 89% of participants reporting they would like to continue treatment and 92% indicating that they would be likely to recommend the treatment to a friend or family member with RLS.

## Discussion

The RESTFUL study, the first large trial to evaluate the efficacy and safety/tolerability of TOMAC for the treatment of medication-refractory RLS, revealed that TOMAC produced significant improvements in RLS symptoms and had excellent safety/tolerability. The CGI-I responder rate (primary endpoint) was almost three-fold higher in the active TOMAC versus sham group. Consistent with clinician ratings of improvement, key secondary endpoints assessed from the participant’s perspective demonstrated that active TOMAC significantly reduced RLS severity, frequency of RLS symptoms, and sleep problems, and improved global symptoms compared with sham. Exploratory analysis indicated that TOMAC reduced trouble falling asleep and daytime sleepiness, common RLS symptoms associated with reductions to quality of life [2]. The reduction in the IRLS total score exceeded the minimally clinically significant difference of 3 points [20], indicating that active TOMAC recipients experienced clinically meaningful improvement. Response to TOMAC was strong regardless of baseline RLS severity; even participants with very severe baseline RLS symptoms showed substantial improvements in response to TOMAC treatment. Device-related AEs were typically limited to mild application site reactions and resolved rapidly with minimal or no follow-up, demonstrating low risk and very good tolerability.

Participants assigned to active TOMAC continued to improve from Week 4 to Week 8, suggesting a trend toward higher response with longer duration use. Participants originally assigned to sham who switched to active TOMAC experienced significant improvement in RLS symptoms during open-label use. For participants assigned to active TOMAC, AE rates decreased from stage 1 to stage 2, indicating a trend towards increased tolerability with longer use. Adherence remained high through 8 weeks and there was no evidence of habituation. Whereas the study duration of 8 weeks was agreed upon with the US FDA as the appropriate duration to establish safety and efficacy for this patient population, the durability of TOMAC response has now been assessed in a separate extension protocol that followed completers of the RESTFUL study for an additional 24 weeks [16].

In contrast to most clinical trials for commonly prescribed RLS medications, this study exclusively recruited medication-refractory patients and did not require medication washout. These differences in methodology could explain the differences in IRLS responses between these clinical trials. For example, influential clinical trials for pramipexole [21] and gabapentin enacarbil [22] reported greater IRLS improvement in response to treatment and placebo than the RESTFUL study showed to TOMAC and sham, respectively. Despite these differences, the effect sizes—measured as the difference in IRLS score between treatment and placebo/sham—were similar across treatments [21, 22], consistent with possibility of similar therapeutic potency. One explanation for the difference in IRLS responses between medication trials and the RESTFUL study is that medication-refractory patients are more resistant to improvement, as would be expected based on their refractory status. Another explanation could be that recent medication washout—common in medication trials but not the RESTFUL study—can result in increased IRLS response to both treatment and placebo. Consistent with this possibility, washout of dopamine agonist medications is thought to cause withdrawal that gradually subsides over weeks to months [23]; the gradual decline of this withdrawal effect could be interpreted as part of the response to treatment (and placebo) in some medication trials.

Since this study specifically assessed medication-refractory RLS, it does not establish the efficacy of TOMAC for patients who are medication-naïve. Subgroup analysis of a previous study suggested that responses to TOMAC may be similar for medication-naïve and medication-refractory RLS [15], and thus further research should be directed at further investigating the medication-naïve patient population. Whereas the blinding assessment results indicate that more participants guessed the correct than the incorrect treatment assignment, guesses were predominantly due to differences in perceived efficacy between active TOMAC and sham, suggesting that correct guesses were primarily attributable to therapeutic efficacy [24] as opposed to limitations in sham design [19].

TOMAC relieves RLS symptoms by engaging similar neuromuscular circuitry as voluntary leg movements such as walking or standing [13], which are known to temporarily relieve RLS symptoms [14]. Our results here demonstrate two fundamental advantages of TOMAC over voluntary movements. First, whereas voluntary movements are not compatible with sleep, TOMAC evoked substantial improvements in sleep quality as measured by the MOS-II and MOS-I. Second, TOMAC provided long-lasting relief for daytime symptoms; the majority of responders (67%) reported relief lasting more than 2 hours after each TOMAC session. In summary, these results suggest that TOMAC simulates the neural signals generated by voluntary leg movements but does so in a manner that provides long-lasting relief, is compatible with sleep, and improves sleep quality.

In summary, TOMAC represents an efficacious and safe treatment option for medication-refractory RLS and is associated with high levels of adherence and patient satisfaction. The safety profile of TOMAC is favorable compared to the recommended medications for RLS [9–12]. Based on its strong efficacy and low risk, TOMAC has the potential to be the leading treatment option for medication-refractory RLS.

## Supplementary Material

zsad190_suppl_Supplementary_Materials_S1

zsad190_suppl_Supplementary_Materials_S2

## References

[1] Allen RP , Picchietti D, Hening WA, Trenkwalder C, Walters AS, Montplaisi J; Restless Legs Syndrome Diagnosis and Epidemiology workshop at the National Institutes of Health. Restless legs syndrome: diagnostic criteria, special considerations, and epidemiology. A report from the restless legs syndrome diagnosis and epidemiology workshop at the National Institutes of Health. Sleep Med. 2003;4(2):101–119. doi:10.1016/s1389-9457(03)00010-8. 14592341

[2] Bogan RK. Effects of restless legs syndrome (RLS) on sleep. Neuropsychiatr Dis Treat. 2006;2(4):513–519. doi:10.2147/nedt.2006.2.4.513. 19412499 PMC2671944

[3] Sabia S , Fayosse A, Dumurgier J, et al. Association of sleep duration in middle and old age with incidence of dementia. Nat Commun. 2021;12(1):2289. doi:10.1038/s41467-021-22354-2. 33879784 PMC8058039

[4] Nagai M , Hoshide S, Kario K. Sleep duration as a risk factor for cardiovascular disease- a review of the recent literature. Curr Cardiol Rev. 2010;6(1):54–61. doi:10.2174/157340310790231635. 21286279 PMC2845795

[5] Pearson VE , Allen RP, Dean T, Gamaldo CE, Lesage SR, Earley CJ. Cognitive deficits associated with restless legs syndrome (RLS). Sleep Med. 2006;7(1):25–30. doi:10.1016/j.sleep.2005.05.006. 16198145

[6] Zhuang S , Na M, Winkelman JW, et al. Association of restless legs syndrome with risk of suicide and self-harm. JAMA Netw Open. 2019;2(8):e199966. doi:10.1001/jamanetworkopen.2019.9966. 31441941 PMC6714009

[7] Ohayon MM , O’Hara R, Vitiello MV. Epidemiology of restless legs syndrome: a synthesis of the literature. Sleep Med Rev. 2012;16(4):283–295. doi:10.1016/j.smrv.2011.05.002. 21795081 PMC3204316

[8] Abetz L , Allen R, Follet A, et al. Evaluating the quality of life of patients with restless legs syndrome. Clin Ther. 2004;26(6):925–935. doi:10.1016/s0149-2918(04)90136-1. 15262463

[9] Silber MH , Buchfuhrer MJ, Earley CJ, Koo BB, Manconi M, Winkelman JW; Scientific and Medical Advisory Board of the Restless Legs Syndrome Foundation. The management of restless legs syndrome: An updated algorithm. Mayo Clin Proc. 2021;96(7):1921–1937. doi:10.1016/j.mayocp.2020.12.026. 34218864

[10] García-Borreguero D , Williams AM. Dopaminergic augmentation of restless legs syndrome. Sleep Med Rev. 2010;14(5):339–346. doi:10.1016/j.smrv.2009.11.006. 20219397

[11] Oertel WH , Hallström Y, Saletu-Zyhlarz GM, Hopp M, Bosse B, Trenkwalder C; RELOXYN Study Group. Sleep and quality of life under prolonged release oxycodone/naloxone for severe restless legs syndrome: An analysis of secondary efficacy variables of a double-blind, randomized, placebo-controlled study with an open-label extension. CNS Drugs. 2016;30(8):749–760. doi:10.1007/s40263-016-0372-1. 27401882 PMC4982896

[12] Trenkwalder C , Beneš H, Grote L, et al.; RELOXYN Study Group. Prolonged release oxycodone-naloxone for treatment of severe restless legs syndrome after failure of previous treatment: a double-blind, randomised, placebo-controlled trial with an open-label extension. Lancet Neurol. 2013;12(12):1141–1150. doi:10.1016/S1474-4422(13)70239-4. 24140442

[13] Charlesworth JD , Adlou B, Singh H, Buchfuhrer MJ. Bilateral high-frequency noninvasive peroneal nerve stimulation evokes tonic leg muscle activation for sleep-compatible reduction of restless legs syndrome symptoms. J Clin Sleep Med. 2023;19(7):1199–1209. doi:10.5664/jcsm.10536. 36856064 PMC10315589

[14] Allen RP , Picchietti DL, Garcia-Borreguero D, et al.; International Restless Legs Syndrome Study Group. Restless legs syndrome/Willis-Ekbom disease diagnostic criteria: updated International Restless Legs Syndrome Study Group (IRLSSG) consensus criteria—history, rationale, description, and significance. Sleep Med. 2014;15(8):860–873. doi:10.1016/j.sleep.2014.03.025. 25023924

[15] Buchfuhrer MJ , Baker FC, Singh H, et al. Noninvasive neuromodulation reduces symptoms of restless legs syndrome. J Clin Sleep Med. 2021;17(8):1685–1694. doi:10.5664/jcsm.9404. 33949942 PMC8656897

[16] Roy A , Ojile J, Kram J, et al. Long-term Efficacy and Safety of Tonic Motor Activation (TOMAC) for Treatment of medication-refractory restless legs syndrome: a 24-week open-label extension study. Sleep. Published online July 13, 2023. doi:10.1093/sleep/zsad188. PMC10566237 37439365

[17] Stevens JE , Mizner RL, Snyder-Mackler L. Neuromuscular electrical stimulation for quadriceps muscle strengthening after bilateral total knee arthroplasty: a case series. J Orthop Sports Phys Ther. 2004;34(1):21–29. doi:10.2519/jospt.2004.34.1.21. 14964588

[18] Rakel BA , Zimmerman BM, Geasland K, et al. Transcutaneous electrical nerve stimulation for the control of pain during rehabilitation after total knee arthroplasty: A randomized, blinded, placebo-controlled trial. Pain. 2014;155(12):2599–2611. doi:10.1016/j.pain.2014.09.025. 25270585 PMC4250415

[19] Rakel B , Cooper N, Adams HJ, et al. A new transient sham TENS device allows for investigator blinding while delivering a true placebo treatment. J Pain. 2010;11(3):230–238. doi:10.1016/j.jpain.2009.07.007. 19945354 PMC2922105

[20] Allen RP. Minimal clinically significant change for the International Restless Legs Syndrome Study Group rating scale in clinical trials is a score of 3. Sleep Med. 2013;14(11):1229. doi:10.1016/j.sleep.2013.08.001. 24051118

[21] Winkelman JW , Sethi KD, Kushida CA, et al. Efficacy and safety of pramipexole in restless legs syndrome. Neurology. 2006;67(6):1034–1039. doi:10.1212/01.wnl.0000231513.23919.a1. 16931507

[22] Lee DO , Ziman RB, Perkins AT, Poceta JS, Walters AS, Barrett RW; XP053 Study Group. A randomized, double-blind, placebo-controlled study to assess the efficacy and tolerability of gabapentin enacarbil in subjects with restless legs syndrome. J Clin Sleep Med. 2011;7(3):282–292. doi:10.5664/JCSM.1074. 21677899 PMC3113968

[23] Buchfuhrer MJ. Strategies for the treatment of restless legs syndrome. Neurotherapeutics. 2012;9(4):776–790. doi:10.1007/s13311-012-0139-4. 22923001 PMC3480566

[24] Hemilä H. Assessment of blinding may be inappropriate after the trial. Contemp Clin Trials. 2005;26(4):512–4; author reply 514. doi:10.1016/j.cct.2005.04.002. 15951244

