# Supplementary material for: Efficacy and safety of tonic motor activation (TOMAC) for medication-refractory restless legs syndrome: a randomized clinical trial
Source: Sleep. 2023 Jul 17;46(10):zsad190. doi: 10.1093/sleep/zsad190 (PMC10566236; doi:10.1093/sleep/zsad190)
Supplement: zsad190_suppl_Supplementary_Materials_S1 [file zsad190_suppl_supplementary_materials_s1.pdf]

Efficacy and Safety of Tonic Motor Activation (TOMAC) for Medication-refractory Restless Legs Syndrome: A Randomized Clinical Trial

Authors:
Richard K. Bogan[1]
Asim Roy[2]
Jerrold Kram[3]
Joseph Ojile[4]
Russell Rosenberg[5]
J. Douglas Hudson[6]
H. Samuel Scheuller[7]
John W. Winkelman[8]
Jonathan D. Charlesworth[9]

Affiliations:
[1]Bogan Sleep Consultants, LLC, Columbia, SC, USA
[2]Ohio Sleep Medicine Institute, Dublin, OH, USA
[3]California Center for Sleep Disorders, San Leandro, CA, USA
[4]Clayton Sleep Institute, LLC, St. Louis, MO, USA
[5]NeuroTrials Research Inc., Atlanta, GA, USA
[6]FutureSearch Trials of Neurology, Austin, TX, USA
[7]Delta Waves, Inc., Colorado Springs, CO, USA
[8]Massachusetts General Hospital, Boston, MA, USA
[9]Noctrix Health, Inc., Pleasanton, CA, USA

Corresponding Author:
Dr. Jonathan D. Charlesworth
Department of Clinical Research
Noctrix Health, Inc.
6700 Koll Center Pkwy, Ste 310
Pleasanton, CA 94566
Phone: (804) 683-4279
jcharlesworth@noctrixhealth.com

CLINICAL INVESTIGATIONAL PLAN

A Multi-Center, Randomized, Double-Blind, Sham-Controlled Study to Evaluate the NTX100 Neuromodulation System for Patients with Medication-Refractory Primary Restless Legs Syndrome (RLS) – The RESTFUL study

**PROTOCOL NUMBER:**	CT-3 (Previously CT-04)
**VERSION:**	2.0 (Superseded Rev A)
**SPONSOR:**	Noctrix Health, Inc.<br>5424 Sunol Blvd<br>Ste 10-451<br>Pleasanton, CA 94566<br>804-683-4279
**SPONSOR REPRESENTATIVE(S):**	Jonathan Charlesworth, PhD<br>Head of Clinical Sciences<br><br>Maggie LeDang<br>Clinical Operations Manager<br><br>Van Lieou<br>Sr. Clinical Project Manager

The information contained in this document is confidential and proprietary property of Noctrix Health, Inc.

Investigator Study Acknowledgement

Read and initial below.

_____ I understand this protocol contains information that is confidential and proprietary to Noctrix Health, Inc.

_____ Any additional information added to this protocol is also confidential and proprietary to Noctrix Health, Inc. and must be treated in the same manner as the contents of this protocol.

_____ I have read the entire protocol.

_____ I understand what the protocol asks me to do as an Investigator.

_____ I will conduct this study following this protocol and will make a reasonable effort to complete the study in the time noted.

_____ I will provide this protocol to study staff under my direct supervision. My study staff will keep the protocol and associated documents confidential.

_____ I will discuss this information with the study staff to ensure they are fully informed about the study and the test articles.

_____ I will not start enrolling in this study until it is approved by a governing Institutional Review Board.

_____ I understand the study may be terminated or enrollment suspended at any time by Noctrix Health, Inc., with or without cause, or by me if it becomes necessary to protect the interests of the study subjects.

____
Name of Investigator

______________________________________ ______________________
Investigator Signature Date

CONFIDENTIAL

All information contained in this document is confidential and is the sole property of Noctrix Health, Inc.

Any reproduction in part or whole without the written permission of Noctrix Health, Inc. is prohibited.

Table of Contents

Investigator Study Acknowledgement 2
Study Design Overview Synopsis 5
Abbreviations 7
1. Background 7
2. Regulatory Status 11
3. Objective 11
4. Study Design 11
5. Blinding 12
6. Duration 13
7. Study population overview 13
7.1. Inclusion criteria (IC) 13
7.2. Exclusion criteria (EC) 14
7.3. Definition of RLS Refractory to Medication (IC #2) 16
8. Investigational Device 17
10. Adverse Events 20
11. Device Tracking 24
12. Device Deficiencies and Malfunctions 24
13. Ethical and Regulatory Considerations 24
13.1. Compliance with Good Clinical Research Practice 24
13.4. Protocol Compliance 26
13.6. Study Monitoring 26
13.7. Safety Reporting 28
13.8. Electronic Case Report Forms/Electronic Data Capture 29
13.9. Quality Assurance Audits 30
13.10. Records Retention 30
13.11. Publication and Reporting of Study Results 30
14. Personnel Responsibilities 31
14.1. Investigator responsibilities: 31

14.2. Sponsor Responsibilities....32
15. Investigator Qualifications....32
16. Study Procedures....32
17. Study Endpoints....40

Study Design Overview Synopsis

**Title**	A Multi-Center, Randomized, Double-Blind, Sham-Controlled Study to Evaluate the NTX100 Neuromodulation System for Patients with Medication-Refractory Primary Restless Legs Syndrome (RLS) – The RESTFUL Study
**Study Device**	NTX100 Neuromodulation System ("NTX100"), an investigational device developed by Noctrix Health, Inc. NTX100 is an external (non-implantable) neurostimulation device that is worn bilaterally on the lower legs.
**Study Objective**	To provide comparative evidence assessing clinically meaningful benefit in the treatment of patients with moderate to severe medication-refractory primary RLS with the NTX100 Neuromodulation System.
**Study Population**	Adults with moderate to severe medication-refractory primary RLS.
**Study Duration**	The treatment period is 8 weeks (56-days)
**Study Interventions**	• Active: NTX100 programmed to **ACTIVE** mode<br>• Sham: NTX100 programmed to **SHAM** mode<br>For each, subjects are instructed to self-administer stimulation on each night that RLS symptoms are present, as needed, to reduce and relieve RLS symptoms. Multiple stimulation sessions may be applied with a maximum of 120 total minutes of stimulation per night.<br>*Subjects who have been prescribed an FDA-approved drug(s) to treat RLS symptoms will be instructed to maintain a constant dose and schedule of this drug(s) throughout the study.*
**Study Design**	The 8 week follow-up duration will consist of two phases:<br>Phase 1: Randomized Double-Blind Phase lasting 4-wks, during which subjects will be randomized 1:1 into Active or Sham study arms<br>Phase 2: Open-Label Phase lasting 4-wks, during which all subjects will receive open-label Active treatment<br>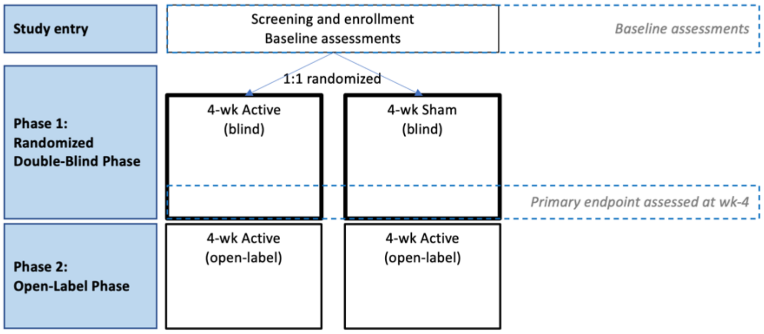

CONFIDENTIAL
All information contained in this document is confidential and is the sole property of Noctrix Health, Inc.
Any reproduction in part or whole without the written permission of Noctrix Health, Inc. is prohibited.

**Maximum Study Duration**	The study is estimated to be completed 18 months after the study opens to enrollment.
**Planned enrollment:**	Approximately 132 subjects will be randomized (66 per Phase 1 study arm). Subjects who meet eligibility criteria will be randomized (1:1) to Active and Sham arms for Phase 1; randomization will be stratified by site at an allocation of 1:1 for the Active and Sham arms.
**Number of Clinical Sites**	4 to 8 clinical sites within the United States.
**Primary Efficacy Endpoint**	The primary outcome measure will be responder rate on the CGI-I scale at Week 4 of Phase 1 relative to Baseline and compared between the study arms.<br><br>Responder rate for the 7-point CGI-I scale will be defined as the proportion of responses of "Much Improved" or "Very Much Improved".
**Key Secondary Efficacy Endpoints**	For all subjects:<br>1. PGI-I responder rate (defined as for CGI-I) at Week 4 of Phase 1, relative to baseline and compared between study arms.<br>2. Mean reduction in IRLS score at Week 4 of Phase 1, relative to Baseline and compared between study arms.<br>3. Mean reduction in MOS-II score at Week 4 of Phase 1, relative to Baseline and compared between study arms.<br>4. Mean reduction in MOS-I score at Week 4 of Phase 1, relative to Baseline and compared between study arms.
**Safety Endpoint**	Descriptive analysis of adverse events for both study arms, classified and tabulated by seriousness, relationship to the device, and severity.
**Additional Study Assessments**	• Daily Questionnaire<br>• Subject characterization<br>• Refractory categorization<br>• Medical history<br>• Concomitant medications<br>• Weekly questionnaire<br>• Custom RLS questionnaire<br>• Blinding assessment

CONFIDENTIAL
All information contained in this document is confidential and is the sole property of Noctrix Health, Inc.
Any reproduction in part or whole without the written permission of Noctrix Health, Inc. is prohibited.

Abbreviations

AE	Adverse Event
AO	Anticipated Observation
CFR	Code of Federal Regulations
CGI-I	Clinical Global Impressions – Improvement
CRF	Case Report Form
DCF	Data Clarification Form
EDC	Electronic Data Capture
FDA	Food and Drug Administration
GCP	Good Clinical Practice
IRB	Institutional Review Board
IRLS	International Restless Legs Syndrome Study Group Rating Scale
IRLSS	International Restless Legs Syndrome Society
ISO	International Organization for Standardization
MOS	Medical Outcomes Study
NPNS	Non-invasive peripheral nerve stimulation
NSR	Non-Significant Risk
PGI-I	Patient Global Impressions – Improvement
QA	Quality Assurance
RLS	Restless Legs Syndrome
SAE	Serious Adverse Event
TENS	Transcutaneous Electrical Nerve Stimulation
USADE	Unanticipated Serious Adverse Device Effect

1. Background

A clinical need has been identified of improved treatment for those suffering with primary idiopathic restless legs syndrome (RLS). Patients with RLS have a strong urge with sensations of tingling/pain, usually in their legs, and often present with a primary complaint of not being able to fall asleep

CONFIDENTIAL
All information contained in this document is confidential and is the sole property of Noctrix Health, Inc.
Any reproduction in part or whole without the written permission of Noctrix Health, Inc. is prohibited.

regularly. This leads to significant quality of life degradation, depression, daytime sleepiness, lack of productivity and a host of downstream effects associated with lack of quality sleep.

1.1. Restless leg syndrome: Background

Restless legs syndrome (RLS) is a sensorimotor disorder that is characterized by a distressing urge to move the legs and in some cases, other parts of the body such as arms [1]. The diagnosis is made by a response to five hallmark identifying criteria instituted by the International Restless Legs Syndrome Society (IRLSS) [2], as quoted below:

> "1. An urge to move the legs usually but not always accompanied by or felt to be caused by uncomfortable and unpleasant sensations in the legs.
>
> 2. The urge to move the legs and any accompanying unpleasant sensations begin or worsen during periods of rest or inactivity such as lying down or sitting.
>
> 3. The urge to move the legs and any accompanying unpleasant sensations are partially or totally relieved by movement, such as walking or stretching, at least as long as the activity continues.
>
> 4. The urge to move the legs and any accompanying unpleasant sensations during rest or inactivity only occur or are worse in the evening or night than during the day.
>
> 5. The occurrence of the above features are not solely accounted for as symptoms primary to another medical or a behavioral condition (e.g., myalgia, venous stasis, leg edema, arthritis, leg cramps, positional discomfort, habitual foot tapping)."

Diagnostically, RLS is considered either primary, often occurring within families, or secondary, developing in association with other conditions (such as iron deficiency anemia, pregnancy or end-stage renal disease).

1.2. Epidemiology

In the United States, RLS is believed to affect more than 10 million adults and an estimated 1.5 million children and adolescents [3]. About one-third of those with RLS symptoms are bothered sufficiently enough to seek medical attention. Epidemiologic studies also show that women are at least 50% more susceptible to RLS than men and RLS is more common in older adults, although it can occur in some as early as the pre-school years.

1.3. Clinical treatments available

CONFIDENTIAL

All information contained in this document is confidential and is the sole property of Noctrix Health, Inc.
Any reproduction in part or whole without the written permission of Noctrix Health, Inc. is prohibited.

The current standard of care involves initial prescription of dopaminergic medications – such as Requip, Mirapex, and Neupro – which initially provide symptomatic relief but often become ineffective over continued usage [4]. Tolerance to these medications is rapid and well-documented [5]; approximately 10% of patients per year become refractory to these medications, and fewer than 20% patients have sustained benefits lasting 10 years or longer [6]. It is also now understood that dopaminergic medications cause what is known as "augmentation", or paradoxical progressive worsening of RLS symptoms that is much faster than the natural progression of the condition. Due to augmentation, patients on dopaminergic medications require increasingly higher doses [7]. Maximal dosage is limited by an increasing risk of side-effects at higher doses, which include compulsive behaviors including substance abuse, hypersexuality, and gambling [8]. As a result of these downsides of dopaminergic agents, a minority of clinicians are starting to prescribe gabapentinioids (e.g. Horizant) as an alternative first-line of treatment; these medications do not typically lead to augmentation but confer risks such as respiratory depression [9], dizziness, and somnolence during the day.

For the large subpopulation of patients who become refractory to dopaminergic medications – typically due to augmentation – there are no FDA approved treatment options and no safe treatment options. As a result of tolerance, augmentation, and dosage limitations, RLS patients often continue to suffer from moderate-severe RLS symptoms while continuing to be reliant on high doses of dopaminergic medications to provide a small degree of relief. To address the massive unmet need, the leading clinicians involved with RLS advocate prescribing off-label opioids [10]. The leading options – oxycodone and methadone, have well documented risks, which include addiction, dependence, overdose, and occasionally death. This situation is especially concerning because primary RLS typically starts in middle age or earlier and persists throughout life, thus patients may end up reliant on opioids for the final decades of their lives.

1.4. Investigational Procedure

The investigational device – the NTX100 Neuromodulation System – is a non-invasive nerve peripheral stimulation (NPNS) device developed by Noctrix Health, Inc. (Sponsor) and designed to bilaterally

All information contained in this document is confidential and is the sole property of Noctrix Health, Inc.
Any reproduction in part or whole without the written permission of Noctrix Health, Inc. is prohibited.

stimulate the common peroneal nerve. Stimulation electrodes are positioned superficially and bilaterally on the lower legs over the head of the fibula bone, a position where the peroneal nerve is closest to the skin. This nerve target innervates regions of the lower extremities commonly associated with RLS symptoms.

1.5 Rationale

The NTX100 Neuromodulation System is a NPNS device that is inspired by the temporary symptomatic relief that RLS patients experience during voluntary movement of their lower extremities. One of the fundamental diagnostic criteria for RLS is the fact that patients report short-lived symptomatic relief during voluntary movement of their legs and feet [2]. This is conceptually similar to the gate-control theory mechanism of pain relief, wherein activation of large sensory fibers suppresses pain signals. However, for RLS, it appears that muscle activation is especially effective – activating sensory fibers of the affected region of the body (e.g. via rubbing or touching) does not typically result in similar relief.

Non-invasive electrical stimulation is known to elicit muscle activation – this feature is used by two types of approved medical devices – powered muscle stimulators (21 CFR 890.5850) and external functional neuromuscular stimulators (21 CFR 882.5810). However, these devices tend to have distracting side-effects including paresthesia and phasic muscle twitches, and thus are incompatible with sleep and inappropriate for use in treating a sleep condition such as RLS. In contrast, the NPNS investigational device tested here produces waveforms that are designed to generate muscle activation without these paresthesia and phasic muscle twitches. Therefore, this technology may be compatible with sleep.

The NTX100 Neuromodulation System stimulates the peroneal nerve at its most superficial position over to the head of the fibula bone to activate muscles of the lower leg including the tibialis anterior at intensity levels that are comfortable and non-distracting. Such comfortable tonic muscle activation is designed to suppress RLS symptoms in a manner similar to voluntary movement – but unlike voluntary movement, may be compatible with sleep. Moreover, the output waveforms and intensities of the NTX100 Neuromodulation System are similar to Functional Electrical Neuromuscular Stimulators and Transcutaneous Electric Nerve Stimulation (TENS) Devices for treatment of pain, two

CONFIDENTIAL
All information contained in this document is confidential and is the sole property of Noctrix Health, Inc.
Any reproduction in part or whole without the written permission of Noctrix Health, Inc. is prohibited.

classes of devices designated nonsignificant risk per 21 CFR 812.3. Therefore, there is a reasonable expectation that the NTX100 Neuromodulation System will lack significant risks.

This study evaluates the effects of NPNS on the symptoms of RLS during in-home subject-administered stimulation. This approach is useful for evaluating safety, usability, tolerability, and preliminary efficacy in a realistic environment - thus identifying any and all barriers to effective and tolerable use.

2. Regulatory Status

The Sponsor has determined that the investigational device in this study – the NTX100 Neuromodulation System – is a nonsignificant risk device under 21 CFR §812.2(b). In previously approved investigational protocols RLS-SNS01 and CT-03, Western IRB confirmed that earlier NTX100 Neuromodulation System prototypes - with comparable stimulation output - were nonsignificant risk devices. Therefore, an approved Investigational Device Exemption (IDE) from FDA is not required to legally perform the study described herein in the US.

The rationale for the nonsignificant risk determination is that the NTX100 Neuromodulation System has equivalent risks and comparable electrical stimulation parameters to Transcutaneous Electric Nerve Stimulation (TENS) Devices for treatment of pain (except for chest pain/angina),” which are Nonsignificant risk (NSR) device per the FDA guidance on Significant Risk and Nonsignificant Risk Medical Device Studies [11].

3. Objective

The study objective is to provide comparative evidence assessing clinically meaningful benefit in the treatment of patients with moderate to severe medication-refractory RLS with the NTX100 Neuromodulation System.

4. Study Design

The study consists of a 4-wk prospective, randomized, sham-controlled, double-blinded phase (Phase 1) followed by a 4-wk prospective open-label phase (Phase 2) for a total of 8-wks of follow-up per subject. The design of the trial is illustrated in the flowchart below (Figure 1).

Phase 1 (4-wks): Subjects who pass screening will be randomly allocated at a 1:1 ratio to receive Active or Sham treatment throughout the 4-wk duration of Phase 1. The primary and key secondary efficacy endpoints of the study will compare the final week of Phase 1 to study entry ("Baseline").

Phase 2 (4-wks): All subjects who complete Phase 1 will be allocated to receive open-label Active treatment throughout the 4-wk duration of Phase 2. Instructions for device use will remain the same as during Phase 1.

Figure 1: Study Design Flowchart

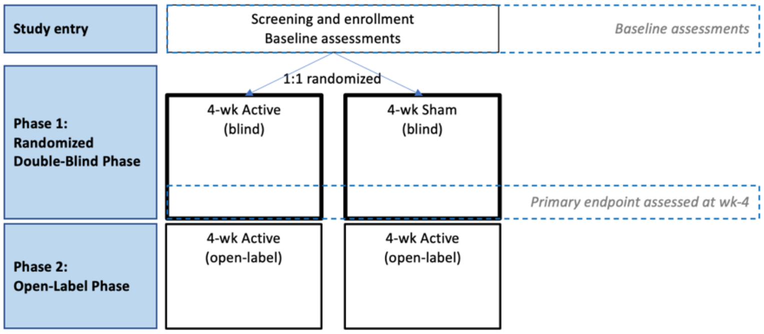

5. Blinding

Phase 1 will be double-blind. Information about treatment assignment will be concealed from subjects, investigators, and site staff, except as required in cases of safety emergencies. In additional, all sponsor staff interacting directly with the research staff or investigators (e.g. in a monitoring capacity) or subjects (e.g. in a device specialist capacity) will be blinded to the Phase 1 treatment assignment throughout the study (even during Phase 2).

Phase 2 will be open-label. At the beginning of Phase 2, the treatment assignment on each device will be set to Active for all subjects. This will take place without revealing the previous treatment assignment during Phase 1.

Active and sham devices will be physically identical and will provide identical visual feedback during operation. Both active and sham mode will have an initial ramp-up in stimulation intensity lasting

CONFIDENTIAL
All information contained in this document is confidential and is the sole property of Noctrix Health, Inc.
Any reproduction in part or whole without the written permission of Noctrix Health, Inc. is prohibited.

approximately 30 seconds and during which sensations are typically noticeable. No subjects with prior neurostimulation experience with NTX100 or any other neurostimulation devices will be enrolled.

The investigator must contact the sponsor if the need to unbreak blinding arises or within 24 hours of an actual unblinding. The Investigator should not open the unblinding envelop UNLESS knowledge of the subject's treatment assignment is required for the subject's clinical care and safety. Documentation of breaking the blind should be recorded in the subjects *Narrative eCRF* with the date and time the blind was broken, and the names of the personnel involved.

6. Duration

The study will be completed approximately 18 months after the study opens to accrual or after 132 subjects have been enrolled, whichever occurs first.

7. Study population overview

The study population will consist of adults with moderate-severe primary RLS. The following inclusion and exclusion criteria are designed to reduce confounding variables and reduce risk.

7.1. Inclusion criteria (IC)

All subjects are required to meet the following inclusion criteria in order to be considered eligible for participation in this study:

1. Subject has received a medical diagnosis of primary restless legs syndrome (RLS).
2. Subject is refractory to RLS medication (as defined in section 7.3).
3. Subject has moderate-severe RLS symptoms as defined by a score of 15 or greater points on IRLS (International Restless Legs Syndrome Study Group Rating Scale [12]) over the week prior to study entry.
4. Subject has RLS symptoms 2 or more nights per week during the week prior to study entry as defined by a score of 2, 3, or 4 on IRLS question #7.
5. RLS symptoms are most significant in the subject's lower legs and/or feet.
6. RLS symptoms are most significant at bedtime, after bedtime, and/or in the 2 hours before bedtime.

CONFIDENTIAL
All information contained in this document is confidential and is the sole property of Noctrix Health, Inc.
Any reproduction in part or whole without the written permission of Noctrix Health, Inc. is prohibited.

7. RLS symptoms between 10am and 6pm are not severe.
8. Subject agrees to not change dosage or schedule of any medications that are known to impact RLS symptoms during the study, including RLS medications, antidepressants, sleep medications, or sedative antihistamines.
9. Subject agrees to not make major lifestyle changes during the study that would significantly affect bedtime, such as major changes to diet, exercise, or career.
10. Subject possesses the necessary equipment, internet/phone accessibility, and communication ability to complete electronic questionnaires and respond to electronic communications and phone calls from the research staff throughout the in-home portion of the study.
11. Subject is ≥ 22 and ≤ 79 years of age when written informed consent is obtained.
12. Subject has signed a valid, IRB-approved informed consent form, can understand the requirements of the study and instructions for device usage, and can converse in English

7.2. Exclusion criteria (EC)

Subjects will be excluded from participating in this study if they meet any of the following exclusion criteria:

1. Subject has RLS that is known to be caused by another diagnosed condition (i.e. secondary RLS).
2. Subject is taking an unstable or inconsistent dose or schedule of medication that is likely to impact RLS symptoms, such as antidepressants, sleep medications, or sedative antihistamines or has changed dosage within the past 30 days.
3. Subject has changed dose and schedule of RLS medications within the month prior to study entry or is otherwise on an inconsistent dose or schedule of RLS medications.
4. Subject has prior experience with neurostimulation devices developed by the study sponsor, has prior experience using neurostimulation devices to treat RLS symptoms, or intends to use a neurostimulation device other than the study device during the study period.
5. Subject was misdiagnosed with RLS, as determined by the investigator (e.g. actual diagnosis of PLMD, arthritis, leg spasms or neuropathy without comorbid RLS).
6. Subject has a primary sleep disorder other than RLS that significantly interferes with sleep at the present time (e.g. obstructive sleep apnea stably controlled via CPAP would not be an exclusion).

CONFIDENTIAL

All information contained in this document is confidential and is the sole property of Noctrix Health, Inc.
Any reproduction in part or whole without the written permission of Noctrix Health, Inc. is prohibited.

7. Subject has active medical device implant anywhere in the body (including but not limited to pacemakers, spinal cord stimulators, deep brain stimulators) or metal implant at the site of study device electrode application.
8. Subject has severe peripheral neuropathy affecting the lower legs and/or subject has neuropathy and is unable to clearly distinguish between symptoms of neuropathy and symptoms of RLS.
9. Subject reports that bedtime is typically outside of 9pm-3am or reports that bedtime regularly varies by more than 4 hours, such as due to shift work.
10. On nights with no RLS symptoms (if any), subject reports typical sleep onset latency of >60min.
11. Subject has been diagnosed with one of the following conditions:
    - Epilepsy or other seizure disorder
    - Current, active or acute or chronic infection other than common cold
    - A malignancy within the past 5 years (not including basal or squamous cell skin cancer)
    - Stage 4-5 chronic kidney disease or renal failure
    - Severe movement disorder symptoms (Parkinson's disease, Huntington's disease, dyskinesia, dystonia)
    - Deep vein thrombosis
    - Multiple sclerosis
12. Subject has moderate or severe cognitive disorder or mental illness.
13. Subject has current diagnosis of iron-deficient anemia or history of iron-deficient anemia within the past year.
14. Subject has known allergy to device materials, electrode gel, polyurethane foam, or lycra (or severe previous reaction to medical adhesives or bandages).
15. Subject has severe edema affecting lower legs.
16. Subject has any of the following at or near the location of device application.
    - Acute injury
    - Cellulitis
    - Open sores

CONFIDENTIAL
All information contained in this document is confidential and is the sole property of Noctrix Health, Inc.
Any reproduction in part or whole without the written permission of Noctrix Health, Inc. is prohibited.

- Other skin condition

17. Subject is on dialysis or anticipated to start dialysis while participating in the study.
18. During the NTX100 calibration process, which is identical for subjects in the active and sham arms, subject reports not feeling stimulation sensations up to an intensity of 30mA, the subject finds stimulation intensities less than 15 mA to be uncomfortable or distracting, or the device does not properly fit the subject.
19. Subject has received another investigational device or drug within 30 days before study entry, is planning to receive another investigational device or drug during the study, or is planning to change RLS medications during the study.
20. Subject has undergone a major surgery (excluding dental work) in the 30 days prior to study entry.
21. Subject is unable or unwilling to comply with study requirements.
22. Subject is pregnant or trying to become pregnant.
23. Subject has a medical condition not listed above that may affect validity of the study as determined by the investigator.
24. Subject has a medical condition not listed above that may put the subject at risk as determined by the investigator.

7.3. Definition of RLS Refractory to Medication (IC #2)

For inclusion criterion #2, the following definition of refractory will be used:

The patient has failed at least one prescription medication administered to treat RLS* for one or more of the following reasons, as determined by the investigator:

1. Adverse effects associated with the medication are intolerable.
2. Patient exhibits symptoms of augmentation.
3. Efficacy has reduced to the point where an up titration would be needed to maintain a sufficient response to medication.
4. The patient lacks sufficient response to medication at the maximum approved or recommended dosage.**

5. The patient lacks sufficient response to medication at the maximum tolerable dosage due to adverse effects.

**Failure of any of the following prescription medications is sufficient to demonstrate that a patient is refractory: Ropinirole (FDA-approved), Pramipexole (FDA-approved), Rotigotine (FDA-approved), Gabapentin enacarbil (FDA-approved), Pregabalin (not FDA-approved for RLS, but administered in clinical practice), Gabapentin (not FDA-approved for RLS, but administered in clinical practice).*

*** per FDA-approved dosage regimen or per RLS consensus statement in the case of off-label medications (Pregabalin or Gabapentin).*

8. Investigational Device

8.1. Description

The NTX100 Neuromodulation System is positioned and worn bilaterally on the legs with stimulation electrodes over the head of the fibula bone, thus targeting the common peroneal nerve – this nerve target innervates regions of the lower extremities commonly associated with RLS symptoms.

For each leg, the NTX100 Neuromodulation System consists of (1) one stimulation unit, (2) two or more electrode areas, and (3) a mechanism for attachment to leg, described below.

- The (1) stimulation unit will:
  - be battery-powered and contain a rechargeable battery and connector for recharging,
  - contain a circuit board that generates the stimulation waveform,
  - contain controls that the subject can use to activate stimulation,
  - contain controls that the subject can use to adjust the intensity of stimulation within a range programmed by the researcher,
  - contain an electronic connection mechanism to deliver a stimulation waveform to the electrodes,
  - contain an interface that the researcher can use to program the range of stimulation intensities, program the mode of stimulation (active or sham), and download data on compliance and functionality.
- The (2) electrodes areas will each:

- be an electrolyte-containing medium or a hydrogel material,
- be disposable, allowing for at least 1 night of use by one subject,
- be attached to the stimulation device by using snaps, magnetic connectors, conductive adhesives, shielded or insulated wires, hook and loop fasteners, or similarly secure connectors,
- have an electrode surface area between 5cm2 and 50cm2.

- The (3) attachment mechanism will:
  - secure the device containing the disposable electrode areas to the body via straps and/or additional biocompatible adhesives.

Description of stimulation parameter ranges allowed by protocol:

- Pulse amplitude: up to 45mA
- Frequency: 2kHz - 6kHz
- Pulse width: 80 - 250 microseconds (depending on frequency)
- Duty cycle: 25-100%
- Pulse shape: Charge-balanced
- Duration: up to 60 minutes per session (up to 120 minutes per day)

8.2. Instructions for Use and Administration

Use of the NTX100 Investigational device is described briefly herein. For details see Instructions for Use.

8.2.1. Route of administration

The NTX100 Neuromodulation System is designed to stimulate the common peroneal nerve; stimulation electrodes within the device are positioned superficially and bilaterally on the lower legs over the head of the fibula bone, a position where the peroneal nerve is close to the skin. This nerve target innervates regions of the lower extremities commonly associated with RLS symptoms.

8.2.2. Dosage and dosage regimen

CONFIDENTIAL
All information contained in this document is confidential and is the sole property of Noctrix Health, Inc.
Any reproduction in part or whole without the written permission of Noctrix Health, Inc. is prohibited.

The dosage regimen depends on the intensity, timing, and duration of stimulation:

1. Intensity. The setpoint stimulation intensity may be the lesser of (a) the maximal intensity that the subject reports is non-distracting and comfortable, as determined in the calibration session described in the instructions for device use and (b) the maximal intensity for each set of stimulation parameters, as determined based on device output capabilities. Subjects may be allowed to adjust stimulation intensity from the setpoint stimulation intensity, but only within a limited pre-programmed range. For example, the subject may increase intensity if symptoms are severe or reduce intensity if symptoms are mild.

2. Timing. Subjects may be instructed to administer stimulation primarily after RLS symptoms become noticeable and before the RLS symptoms become severe.

3. Duration. Each session of stimulation may last 30 minutes. Stimulation may be activated for up to 120 minutes per day (e.g., up to 4 x 30-minute sessions), depending on the timing, duration, and severity of RLS symptoms for the specific subject.

8.3. Contraindications

The following are contraindications to device usage:

- Diagnosis of epilepsy or other seizure disorder,
- Active medical device implant anywhere in the body, including but not limited to pacemakers, spinal cord stimulators, deep brain stimulators
- Metal implant at the site of study device application
- Known allergy to device materials (or severe previous reaction to medical adhesives or bandages)
- Cellulitis, open sores, or acute injury at or near the location of device electrode application

9. Risks

9.1. Investigational device risks

NPNS devices such as NTX100 Neuromodulation System result in a known potential for the following anticipated observations, which are typically mild to moderate, transient in nature, and resolve over time.

CONFIDENTIAL
All information contained in this document is confidential and is the sole property of Noctrix Health, Inc.
Any reproduction in part or whole without the written permission of Noctrix Health, Inc. is prohibited.

- Mild skin irritation from use of adhesive electrodes and/or secondary attachment mechanism.
- Discomfort, paresthesia, or otherwise irritating or uncomfortable sensations during active electrical stimulation.
    - *This risk is reduced by calibrating the stimulation intensity.*
- Temporary interference with sleep while wearing the device:
    - For some individuals, device may be uncomfortable, thus interfering with sleep while wearing the device.
    - For some individuals, this device may interfere with preferred sleep positions, thus interfering with sleep during usage.
- Temporary increase in RLS symptoms while wearing the device:
    - For some individuals, this device may interfere with voluntary leg movements used to relieve RLS symptoms, thus leading to a temporary increase in RLS symptoms.
    - In some cases, this device may otherwise lead to a temporary increase in RLS symptoms during active stimulation for other reasons.
        - *This risk may be reduced by calibrating the stimulation intensity and/or adjusting the schedule of stimulation.*

10. Adverse Events

Anticipated observations (e.g. common treatment effects) and adverse events occurring during the study will be recorded. Descriptions of anticipated observations and AEs will include the date of onset, the date it ended, the severity, the relationship to study device, and the outcome. All reported AEs will be summarized by the number of subjects reporting AEs, system organ class (where applicable), severity, seriousness, and relationship to study device.

The study Investigator and Coordinator will evaluate, characterize and record in the electronic Case Report Form (eCRF) all adverse events (AEs) occurring in all subjects from the time of enrollment to study exit (or premature withdrawal). Adverse events unresolved at study exit will be followed by the

CONFIDENTIAL
All information contained in this document is confidential and is the sole property of Noctrix Health, Inc.
Any reproduction in part or whole without the written permission of Noctrix Health, Inc. is prohibited.

Investigator until resolution occurs or at least 60 days after the subject's participation in the study is complete. AEs may be reported spontaneously by the subject or detected by the Investigator or Coordinator. AEs should be evaluated for diagnoses not just symptoms (i.e., "angina", not "chest pain").

In addition to verbatim terms, the Sponsor may categorize all AEs using MedDRA preferred terms (PT) and system organ classes (SOC). Analysis may report both verbatim and MedDRA terms.

10.1. Adverse Event Definitions

An **adverse event** (AE) is any untoward medical occurrence, independent of its association with the investigational device. AEs also include any adverse laboratory signs or physical exam findings.

A **serious adverse event** (SAE[1]) is any AE that:

- led to a death,
- led to a serious deterioration in the health of the subject that:
  - resulted in a life-threatening illness or injury,
  - resulted in a permanent impairment of a body structure or a body function,
  - required in-patient hospitalization or prolongation of existing hospitalization,
  - resulted in medical or surgical intervention to prevent permanent impairment to body structure or a body function
- or led to fetal distress, fetal death or a congenital abnormality or birth defect.

A **device-related SAE** is an event meeting the SAE definition above that is also rated as probably or definitely related the investigational device. No device-related SAEs have been reported in prior studies. Note that an elective or pre-planned hospitalization for a condition that did not worsen during the study is not an AE.

An **unanticipated serious adverse device effect** (USADE) is any SAE that is caused by, or associated with, a device, if that effect, problem, or death was not previously identified in nature, severity, or degree of incidence in the investigational plan or application (including a supplementary plan or application).

[1] Definition from ISO14155:2011

All information contained in this document is confidential and is the sole property of Noctrix Health, Inc.
Any reproduction in part or whole without the written permission of Noctrix Health, Inc. is prohibited.

10.2. AE Severity and Relatedness

Each AE occurring in the study will be characterized by the study Investigator as to severity (Table 1) and relatedness (Table 2).

Table 1. AE Severity Grading System.

Grade	Brief description
Grade 1	Mild; asymptomatic or mild symptoms; clinical or diagnostic observations only; intervention not indicated
Grade 2	Moderate; minimal, local, or non-invasive intervention indicated; limiting age-appropriate instrumental ADL[a]
Grade 3	Severe or medically significant but not immediately life-threatening; hospitalization or prolongation of hospitalization indicated; disabling; limiting self-care ADL[b]
Grade 4	Life-threatening consequences; urgent intervention indicated
Grade 5	Death related to adverse event

[a]'Instrumental ADL' refers to activities of daily living such as preparing meals, shopping for groceries or clothes, using the telephone, and managing money. [b]'Self-care ADL' refers to bathing, dressing and undressing, feeding oneself, using the toilet, taking medications, and not being bedridden. From the National Cancer Institute Common Terminology Criteria for Adverse Events v4.0 NCI, NIH, DHHS. May 29, 2009 NIH publication #- 09-7473.

Table 2. AE Relatedness Grading System.*

Grade	Relationship of AE to study device	Description
5	Definite	An event that follows a reasonable temporal sequence from administration of the study device; that follows a known or expected response pattern to the study device; and that is confirmed by improvement on stopping.

4	Probable	An event that follows a reasonable temporal sequence from administration of the study device; that follows a known or expected response pattern to the study device; and that is unlikely to have been caused by concurrent/underlying illness or other drugs, procedures, or other causes.
3	Possible	An event that follows a reasonable temporal sequence from administration of the study device; that follows a known or expected response pattern to the study device; but may have been caused by concurrent/underlying illness, drugs, procedure, or other causes.
2	Unlikely	An event that does not follow a reasonable temporal sequence from administration of the study device; that does not follow a known or expected response pattern to the study device, or most likely was caused by concurrent/underlying illness, drugs, procedure, or other causes, because of their known effects.
1	Not related	An event almost certainly caused by concurrent/underlying illness, drugs, procedure, or other causes.

** AEs occurring before treatment with the study device will be categorized as unrelated to the study device.*

10.3. Adverse Event Reporting

Investigators must report all SAEs to the study Sponsor and governing IRB within 3 business days or according to local IRB guidelines. Investigators should call the study sponsor immediately upon becoming aware of the occurrence of an SAE. The sponsor will contact the independent medical reviewer, to assist in assessing any safety concerns, if needed. The Investigator should be able and willing to provide further information on the specific event when requested by the study Sponsor. If the Investigator learns of an SAE that occurs within 1 month after the subject completes the study, he/she should notify the Sponsor. Investigators must also report all AEs to the governing IRB as determined by that IRB.

Prompt AE evaluation:

- protects the safety of study subjects;
- aids in understanding the overall safety profile of the device;
- prompts, if necessary, modification to the study protocol

CONFIDENTIAL
All information contained in this document is confidential and is the sole property of Noctrix Health, Inc.
Any reproduction in part or whole without the written permission of Noctrix Health, Inc. is prohibited.

- allows improvements in study design or procedures; and
- adheres with standard good clinical practices.

11. Device Tracking

The Sponsor will send the investigational devices to the Investigator. The Investigator must house study devices in a secure location.

The Investigator must carefully and completely track receipt, use and disposition of all investigational devices. The Sponsor will track sending and receiving of devices. The Sponsor will monitor site device accountability periodically.

If a Sponsor representative or designee is present at the time of use, he/she may directly take possession of used device(s). All devices will be returned to the Sponsor after the study is complete.

12. Device Deficiencies and Malfunctions

Throughout the study, the Investigator and study staff will report and document all device deficiencies and malfunctions related to the identity, quality, durability, reliability, safety or performance of the device. This includes reporting of device deficiencies/malfunctions that did not lead to an AE but could have if: 1) suitable action had not been taken, 2) intervention had not been made, or 3) circumstances had been less fortunate. If possible, the Investigator should return devices suspected of deficiency or malfunction to the Sponsor for analysis.

13. Ethical and Regulatory Considerations

13.1. Compliance with Good Clinical Research Practice

This study will be conducted in compliance with the principles of the Belmont Report, the Declaration of Helsinki, with the current Good Clinical Practice (GCP) guidelines and with other applicable regulations. The Investigator and all study staff will conduct the study in compliance with this protocol. Voluntary informed consent will be given by every subject prior to the initiation of any study-related procedures. The rights, safety and well-being of the study subjects are the most important considerations and prevail over the interests of science and society. All personnel

involved in the conduct of this study must be qualified by education, training and experience to perform their assigned responsibilities.

13.2. Confidentiality of Data

All information and data sent to the Sponsor, Contract Research Organizations, or the Independent Medical Reviewer concerning subjects or their participation in this study will be considered confidential. All data used in the analysis and reporting of this evaluation will be used in a manner without identifiable reference to the subject. The investigator consents to visits by the staff of the Sponsor and its authorized representatives and the U.S. Food and Drug Administration or any other governmental body to review the study subjects' medical records including any test or laboratory data.

13.3. Institutional Review Board (IRB) and Informed Consent

Before study initiation, the Investigator must have written and dated approval from the IRB for the protocol, consent form, subject recruitment materials/process (e.g., advertisements), and any other written information to be provided to subjects. The Investigator should also provide the IRB with a copy of the product labeling, information to be provided to subjects and any updates. The Investigator will submit documentation of the IRB approval to the Sponsor. Copies of all correspondence with the IRB regarding this study must be sent to the Sponsor.

The IRB-approved consent form must include all elements required by FDA, state, and local regulations, and may include appropriate additional elements.

The Investigator/designee will explain the study to each potential subject and the subject must indicate voluntary consent by signing and dating the approved informed consent form. The Investigator must provide the subject with a copy of the consent form in a language the subject understands. The Investigator will maintain documentation that informed consent was obtained prior to the initiation of any study-specific procedures.

CONFIDENTIAL
All information contained in this document is confidential and is the sole property of Noctrix Health, Inc.
Any reproduction in part or whole without the written permission of Noctrix Health, Inc. is prohibited.

Withdrawal of IRB approval of the Investigator's part in the investigation must be reported to the Sponsor within 5 working days.

13.4. Protocol Compliance

The Investigator will comply to the extent possible with the IRB-approved protocol. All deviations from the protocol must be documented. The Investigator will notify the Sponsor immediately if a deviation from the protocol was required to protect subject safety.

13.5. Protocol Revisions

Revisions to the study protocol can be made only by the study Sponsor. A revised protocol can be put into place only after governing IRB approval. All administrative letters must be submitted to the IRB for their information.

New or altered consent forms required by the IRB due to a protocol change must be signed by all subjects currently enrolled in the study and must be used for any subsequent subject enrollment.

13.6. Study Monitoring

Representatives of the Sponsor will visit all study sites to perform monitoring and data management functions, and provide participating sites with relevant contact information, as necessary. Study monitors may change periodically over the course of this study. All monitors will be qualified to perform their assigned responsibilities, and participating investigators/site personnel will be notified of any changes as they occur. All Study monitors will be blinded to Phase 1 treatment allocation.

On-site and/or remote monitoring of all participating sites will be frequent enough to assure continued acceptability of the data by assessing site compliance with the study protocol, adherence to data collection procedures, and maintenance of study records. Scheduled site visits will include, but are not limited to, the following:

- Site initiation visit: prior to enrolling subjects, an on-site or remote initiation visit will be conducted by clinical study personnel to review this study protocol and discuss eCRF completion and transmittal procedures. Alternatively, a meeting may be conducted for several sites at a common location.

- Interim monitoring site visit: on-site and/or remote monitoring visits will be conducted at all sites to assess the progress of the study and identify any concerns that result from review of the study records, study management documents, or subject informed consent documents. To assure the integrity of the data, a representative number of individual subject records and other supporting documents will be compared to eCRFs completed at the site to determine that:

    - The study protocol is being followed, and only eligible subjects are being enrolled; variances, if they occur, are recorded and reported as appropriate.
    - Informed consent is properly documented.
    - Adverse events are being reported appropriately.
    - Information recorded on eCRFs is complete, accurate and legible.
    - Subjects failing to complete the clinical study and the reason for failure are properly recorded.

- Final monitoring/Close-out site visit: a final visit to participating sites may be made by the study monitor, if necessary. Any ongoing responsibilities will be discussed with the investigator and/or site personnel as appropriate.

At the close of the study at an investigational site, appropriately trained personnel appointed by the Sponsor will perform a close-out process via the telephone or on-site. The purpose of this visit is to collect all outstanding study data documents, ensure that the investigator's files are accurate and complete, review record retention requirements, ensure final accounting of all investigational devices shipped to the investigator, provide for appropriate disposition of any remaining supplies,

CONFIDENTIAL
All information contained in this document is confidential and is the sole property of Noctrix Health, Inc.
Any reproduction in part or whole without the written permission of Noctrix Health, Inc. is prohibited.

and ensure that all applicable requirements are met for the study. The observations and actions made during the intervention will be documented and communicated to the investigator.

Representatives of government regulatory authorities may also evaluate the study records, source documents, Investigator, study staff and facilities.

The Investigator should immediately notify the Sponsor of any audits of this study by any regulatory agency, and must promptly provide copies of any audit reports.

13.7. Safety Reporting

The Sponsor is responsible for ongoing safety evaluation in this study protocol. Sponsor activities regarding safety include:

- classification of all AEs
- review of all AEs reported in the study
- confirm site's classification of AEs in terms of severity and relatedness to the study device
- review of severity and relatedness with the study Investigator, especially when there is disagreement between the Investigator and the sponsor
- review of device deficiencies and malfunctions, including determination and documentation of whether deficiencies/malfunctions could have led to an SAE
- ensuring the reporting of all SAEs and device deficiencies/malfunctions that could have led to an SAE to the IRB and, if required, regulatory authorities in a timely fashion
- informing all site Investigators in writing of all SAEs at all sites in a timely fashion
- updating the risk analysis and assessment of corrective or preventive actions potentially required as a result of new information obtained in the investigation

The Sponsor will evaluate all serious adverse events against US reporting requirements (Medical Device Reporting, 21 CFR 812) and Medical Device Directive (vigilance incident reporting) as per its standard operating procedures. The Sponsor will investigate each SAE to determine whether the event represents an unanticipated serious adverse device effect (USADE, see Section 7). The

All information contained in this document is confidential and is the sole property of Noctrix Health, Inc.
Any reproduction in part or whole without the written permission of Noctrix Health, Inc. is prohibited.

Sponsor will report any event to regulatory authorities, Investigators and reviewing IRBs/ECs as necessary. If an investigation shows that a USADE presents an unreasonable risk to subjects, the Sponsor will terminate all investigations or parts of investigations presenting that risk as soon as possible. The Sponsor will only resume a terminated investigation after corrective actions have taken place, site Investigators are informed and IRBs/ECs have been notified and given approval to resume the study.

13.8. Electronic Case Report Forms/Electronic Data Capture

The study will use an electronic data capture (EDC) system to implement electronic case report forms (eCRF). The system will allow compliance with 21 CFR 11 Electronic Signatures. All CRF's are housed in the EDC system. The Investigator and Coordinator will be trained in use of the eCRF prior to study initiation. Retraining in use of EDC can occur at any time. The EDC system will be validated prior to use and after any modification is made. Each eCRF will be designed to accommodate the specific features of the trial design. Modification of a eCRF will only be made if deemed necessary by the study sponsor.

An eCRF is required and should be completed for each included subject. The Investigator has ultimate responsibility for the collection and reporting of all data entered on the eCRFs and any other data collection forms (source documents) and ensuring that they are accurate, authentic/original, attributable, complete, consistent, legible, timely (contemporaneous), enduring and available when required. The eCRFs must be signed by the investigator to attest that the data contained therein are true.

The site will be provided with general eCRF Completion Guidelines which will assist in data entry and data issues/questions. All persons allowed to enter or change eCRF data must appear on the Delegation of Responsibilities Log.

CONFIDENTIAL
All information contained in this document is confidential and is the sole property of Noctrix Health, Inc.
Any reproduction in part or whole without the written permission of Noctrix Health, Inc. is prohibited.

The Sponsor will remotely monitor the eCRFs to identify possible data errors. The system will have a query mechanism whereby the site Coordinator can respond to Sponsor queries. All data discrepancies will be resolved prior to database lock.

13.9. Quality Assurance Audits

Sponsor representatives or designees may conduct site quality assurance (QA) audits during the study. The Investigator must agree to provide the auditor with direct access to all relevant documents and discuss any findings with the auditor.

In the event of an inspection by the FDA or other regulatory authorities, the Investigator must give the inspector direct access to relevant documents and to discuss any findings with the inspector. The Investigator must notify Sponsor in the event of a FDA site audit.

13.10. Records Retention

The Investigator must maintain all study records (including device disposition, informed consents, source documents, correspondence, regulatory documents, contracts etc.) for at least 2 years after study completion. At the Investigator's discretion, all records may be sent to the Sponsor for permanent storage.

The Investigator must contact the Sponsor or designee prior to destroying any records associated with this study. If the Investigator withdraws from the study, all study-associated records must be transferred to a mutually agreed upon designee. Written notification of such a transfer must be given to the Sponsor or designee.

13.11. Publication and Reporting of Study Results

The study will be registered with clinicaltrials.gov before the first subject is enrolled. Study results will be documented in a study report that will be signed by Sponsor representatives and by each Investigator who enrolls subjects in the study, unless otherwise noted.

CONFIDENTIAL
All information contained in this document is confidential and is the sole property of Noctrix Health, Inc.
Any reproduction in part or whole without the written permission of Noctrix Health, Inc. is prohibited.

If the results of this study will be published, all standard editorial and ethical practices, will be followed. Results from multi-center studies must be published or presented at congresses only in their entirety with data pooled from all centers. Individual Investigators may not publish data from individual centers, unless granted specific written permission from the Sponsor to do so.

The list of authors of any formal publication or presentation of study results may include, as appropriate, representatives of the Sponsor.

14. Personnel Responsibilities

14.1. Investigator responsibilities:

Investigators are responsible for ensuring the investigation is conducted according to all signed agreements, the study protocol, and applicable regulatory agency regulations (21 CFR 812), which include:

a) Permit monitor inspection of facilities and records.
b) Permit FDA and other government health authorities' inspection of facilities and records.
c) Submit protocol and informed consent to IRB and await approval.
d) Submit proposed amendments to protocol and informed consent to IRB and await approval, unless the change reduces the risk to subjects.
e) Obtain informed consent of subjects.
f) Implement study in accordance with protocol.
g) Complete case report forms.
h) Record and explain deviations from protocol and report to monitor.
i) Submit annual progress reports, final reports, and adverse effect reports to IRB and sponsor.
j) Record the receipt, disposition, and return of study devices.
k) Refrain from promoting study or study articles in such a way that the potential subject will be biased in his/her responses.
l) Maintain medical histories of subjects.

CONFIDENTIAL
All information contained in this document is confidential and is the sole property of Noctrix Health, Inc.
Any reproduction in part or whole without the written permission of Noctrix Health, Inc. is prohibited.

m) Retain records for two years following FDA approval of marketing application.

14.2. Sponsor Responsibilities

Listed below are the Sponsor's responsibilities for this study.

a) Assure IRB approval of protocol and informed consent is obtained.

b) Select and train monitors.

c) Select investigators.

d) Train investigators in device use.

e) Obtain Agreement Letter and curriculum vitae and proof of appropriate licensure of investigator and other study staff.

f) Control shipment of investigational devices.

g) Conduct day-to-day administration of study.

h) Investigate unanticipated, device related adverse effects.

i) Document protocol deviations and violations.

j) Obtain statement of financial disclosure.

k) Will appoint trained and qualified representative(s) to perform initial pre-screening.

l) May supervise device calibration.

15. Investigator Qualifications

Investigators must have an active license and board certification and experience in the treatment of RLS, as documented on their Curriculum Vitae and/or in a statement of the investigator's relevant experience, including dates, location, extent, and type of experience. Each Investigator must undergo training conducted by the Sponsor, on the study device prior to study initiation.

16. Study Procedures

16.1. Overview

The study consists of 8-weeks of in-home treatment, consisting of a 4-wk sham-controlled phase (Phase 1) followed by a 4-wk open-label active treatment phase (Phase 2). In-person visits are scheduled at the start and end of each phase (Days 0, 28, 56) and at Day 14. Remote follow-ups –

CONFIDENTIAL
All information contained in this document is confidential and is the sole property of Noctrix Health, Inc.
Any reproduction in part or whole without the written permission of Noctrix Health, Inc. is prohibited.

calls and/or video calls – are scheduled at additional timepoints throughout both phases to assess interim response to treatment, as discussed in Section 16.13 below.

16.2. Recruitment

Among applicants, subject eligibility will be determined based on the inclusion and exclusion criteria discussed above. A pre-screening call will be completed prior to the first in-person visit (and thus prior to consent) to exclude most ineligible applicants prior to the in-person visit. The pre-screening call will be performed by a sponsor-appointed representative. Eligibility will be contingent on scheduling constraints, such as research facility and research staff availability.

16.3. Informed consent Process

All subjects must be provided a consent form describing the study with sufficient information for subjects to make an informed decision regarding their participation. Subjects must sign the IRB approved informed consent prior to participation in any study specific procedure, with the exception of pre-screening. The subject must receive a paper or electronic copy of the signed and dated consent document. The signed copy of the consent document must be retained in the study binder in paper or electronic format. The schedule of visits and procedures is provided in Table 3.

16.4. Screening

Potential subjects will be screened for eligibility based on the criteria in Section 7 above. Device calibration (Section 16.5 below) will be part of the screening process, consistent with Exclusion Criterion #18. A potential subject who signs the study consent and is eligible will be assigned a study ID number, will be considered enrolled, and will count towards the study's sample size. Potential subjects who fail Exclusion Criterion #18 and experience an adverse event (AE) during device calibration will be assigned a study ID for the purpose of tracking this AE but will not be randomized to a treatment arm and will not count towards the study's sample size.

16.5. Device calibration

During screening, the stimulation intensity of the investigational device will be calibrated separated for each leg (left and right), using the demo calibration kit provided by the sponsor. If the subject does not fail Exclusion Criterion #18 during calibration the subject will be randomized within the EDC system, the calibrated values will be programmed into the investigational device kit that corresponds

CONFIDENTIAL
All information contained in this document is confidential and is the sole property of Noctrix Health, Inc.
Any reproduction in part or whole without the written permission of Noctrix Health, Inc. is prohibited.

to the randomization number generated. The calibration process is designed to identify the maximally effective settings that are comfortable for each subject. The sponsor may administer the calibration and/or assist in administering the calibration.

16.6. Baseline Assessments

At study entry, the following assessments are completed: IRLS [12], Subject characterization, Medical history, Refractory characterization, MOS-Sleep (Medical Outcomes Study Sleep Scale [13]), and Concomitant medications. If the screening process takes multiple days, the IRLS and MOS-Sleep must be administered on the final day of screening.

16.7. Randomization

Following consent and enrollment, authorized site staff will be provided with each subject's assigned study device kit number by the EDC system during the randomization process. The site staff will record the assigned randomization number on both the device kit label and the subject's source documents. The site staff will also record the randomization assignment in the Device Accountability Log. Subjects will be randomized in a 1:1 to either:

- NTX100 programmed to **ACTIVE** mode
- NTX100 programmed to **SHAM** mode

Randomization assignments will be stratified by study center with randomly chosen block sizes of 4 or 6. Small but randomly determined block sizes preserve treatment assignment balance within study center while maintaining assignment unpredictability. Any Investigator who is discovered to tamper with randomization will be immediately terminated from the study.

16.8. Device training

Following randomization, subjects are trained on proper usage of the device, including positioning of the devices on their legs. Instrument(s) designed for marking the human skin (body-marking pen or temporary tattoo) may be employed to mark the locations of electrode placement. Device calibration may be adjusted during device training based on subjective feedback from the subject.

16.9. Transition to open-label active treatment

CONFIDENTIAL
All information contained in this document is confidential and is the sole property of Noctrix Health, Inc.
Any reproduction in part or whole without the written permission of Noctrix Health, Inc. is prohibited.

At Day 28, all subjects will receive NTX100 programmed to **ACTIVE** mode for use throughout Phase 2 and will be instructed that they will now be receiving Active treatment. Neither the research staff nor the subjects are retrospectively informed of the mode used during Phase 1.

16.10. Study Treatment Description

Study subjects are instructed to self-administer treatment nightly as needed – typically after RLS symptoms start and before RLS symptoms become severe. The instructions for timing of device use may be adjusted based on the timing of RLS symptoms experienced by the subject. Once activated, a single session of treatment may run for approximately 30 minutes and then turn off automatically. Up to 120 total minutes per night may be used. If device battery life limits the number of uses per night, the following two timings of use may be prioritized: (1) at bedtime, to reduce symptoms and thus help with sleep initiation, and (2) when waking up in the middle of the night, to reduce symptoms and thus help with sleep re-initiation. Device intensity is typically set to the calibrated levels, but intensity may be increased if RLS symptoms are especially severe or decreased such as if RLS symptoms are mild.

16.11. Concomitant Medications and Treatments

Study subjects are instructed to maintain a stable dose and schedule of all concomitant medications and treatments for RLS and of all concomitant medications and treatments that affect RLS symptoms, including antidepressants, sleep medications, or sedative antihistamines. Concomitant medications and treatments are tracked throughout the study based on subject reported data.

16.12. Leg movement data collection

The NTX100 Neuromodulation System may have the capability to collect data on leg movements, such as by using accelerometers and/or gyroscope sensors, while the subject is wearing the devices. These data may be collected during this study and used for subsequent analysis and development.

16.13. Evaluation and study assessment schedule

The following evaluation calls and/or visits are scheduled, as outlined in Table 3 below. A window of +/- 2 days may be utilized on office follow-ups and a window of +/- 1 day for any phone follow-up, as needed.

CONFIDENTIAL
All information contained in this document is confidential and is the sole property of Noctrix Health, Inc.
Any reproduction in part or whole without the written permission of Noctrix Health, Inc. is prohibited.

Phase 1 -

Evaluation 1: **Office** visit for enrollment, including consent, screening, device calibration, randomization and training on device usage. This may involve a single visit or multiple visits.

Evaluation 2: (3-days after Eval. #1) Phone follow-up to assess NTX100 use and assess the occurrence of adverse events.

Evaluation 3: (7-days after Eval. #1) Phone follow-up to assess RLS symptoms, NTX100 use and assess the occurrence of adverse events.

Evaluation 4: (14-days after Eval #1) **Office** follow-up to assess RLS symptoms, NTX100 use, and assess the occurrence of adverse events and/or changes to daily medications.

Evaluation 5: (21-days after Eval. #1) Phone follow-up to assess RLS symptoms, NTX100 use and assess the occurrence of adverse events.

Evaluation 6: (28-days after Eval #1) **Office** follow-up to assess RLS symptoms, NTX100 use, and assess the occurrence of adverse events and/or changes to daily medications.

Phase 2 -

Evaluation 7: (3-days after Eval. #6) Phone follow-up to assess NTX100 use and assess the occurrence of adverse events.

Evaluation 8: (7-days after Eval. #6) Phone follow-up to assess RLS symptoms, NTX100 use and assess the occurrence of adverse events.

Evaluation 9: (14-days after Eval #6) Phone follow-up to assess RLS symptoms, NTX100 use, and assess the occurrence of adverse events and/or changes to daily medications.

Evaluation 10: (21-days after Eval. #6) Phone follow-up to assess RLS symptoms, NTX100 use and assess the occurrence of adverse events.

Evaluation 11: (28-days after Eval #6) **Office** follow-up to assess RLS symptoms, NTX100 use, and assess the occurrence of adverse events and/or changes to daily medications.

Evaluations 1, 4, 6, and 11 are intended to be in-person and all other Evaluations are designed to be remote interactions (calls or video calls). Evaluation 4 may be replaced by remote interactions to the extent needed to reduce risk to subjects and/or to the extent that technology allows for remote interactions.

CONFIDENTIAL

All information contained in this document is confidential and is the sole property of Noctrix Health, Inc.
Any reproduction in part or whole without the written permission of Noctrix Health, Inc. is prohibited.

The schedule of assessments are shown in Table 3 below. In addition to these assessments, subjects will complete a Daily Questionnaire each day throughout the 8-wk study duration. Additionally, the devices may automatically log quantitative compliance, usage data, and leg movement data throughout the duration of the study.

TABLE 3: SCHEDULE OF ASSESSMENTS

	**EVAL 1**	EVAL 2	EVAL 3	**EVAL 4**	EVAL 5	**EVAL 6**	EVAL 7	EVAL 8	EVAL 9	EVAL 10	**EVAL 11**
	Day 0* *in-office*	Day 3	Day 7	Day 14 *in-office*	Day 21	Day 28 *in-office*	Day 31	Day 35	Day 42	Day 49	Day 56 *in-office*
Informed Consent	X										
Screening	X										
Subject characterization	X										
Medical History	X										
Refractory categorization	X										
Concomitant Medications	X			X		X			X		X
Randomized receipt of Treatment or Sham Device	X										
Begin open-label use of Treatment Device						X					
Daily Questionnaire follow-up		X	X	X	X	X	X	X	X	X	X
Weekly questionnaire and follow-up			X	X	X	X		X	X	X	X
CGI-I						X					X
PGI-I				X		X			X		X
IRLS	X			X		X			X		X
MOS-Sleep	X					X					X
Custom RLS questionnaire						X					X
Blinding Assessment						X					
Adverse Events		X	X	X	X	X	X	X	X	X	X

** If EVAL 1 spans multiple days, Day 0 refers to the final day of EVAL 1.*

16.14. Administration of assessments

- **PGI-I** (Patient Global Impressions – Improvement Scale). The subject and not the examiner (site staff) should make the ratings, but the examiner should be available to clarify any misunderstandings the subject may have about the questions. The examiner should mark the subject's answers on the form.
- **CGI-I** (Clinical Global Impressions – Improvement Scale). The CGI-I is completed by the Investigator after reviewing the subject's PGI-I response and conducting a short interview with the subject regarding their RLS symptoms over the past week compared to baseline. The Investigator will not be permitted access to the subject's IRLS responses.

CONFIDENTIAL
All information contained in this document is confidential and is the sole property of Noctrix Health, Inc.
Any reproduction in part or whole without the written permission of Noctrix Health, Inc. is prohibited.

- **Refractory categorization.** The refractory categorization form should be completed by the Investigator, within 72 hours of EVAL 1.
- **IRLS.** The subject and not the examiner (site staff) should make the ratings, but the examiner should be available to clarify any misunderstandings the subject may have about the questions. The examiner should mark the subject's answers on the form.
- **MOS-Sleep.** The subject and not the examiner (site staff) should make the ratings, but the examiner should be available to clarify any misunderstandings the subject may have about the questions. The examiner should mark the subject's answers on the form.
- **Blinding assessment**. The subject and not the examiner (site staff) should make the ratings, but the examiner should be available to clarify any misunderstandings the subject may have about the questions. The examiner should mark the subject's answers on the form.
- The **Daily Questionnaire** will be completed by the subject.
- **Subject characterization** will be completed by the examiner (site staff) based on information from the subject.
- **Medical history** will be completed by the examiner (site staff) based on information from the subject.
- For the **Weekly questionnaire**, the subject and not the examiner (site staff) should make the ratings, but the examiner should be available to administer the questions and clarify any misunderstandings the subject may have about the questions. The examiner should mark the subject's answers on the form.

16.15. Study Exit

Study exit will occur at the conclusion of Eval #11. At study exit, the Investigator/coordinator will complete the study exit eCRF. Adverse events unresolved at study exit will be followed by the Investigator until resolution occurs or at least 60 days after the subject's participation in the study is complete.

16.16. Subject Discontinuation

A subject may be removed from the study prior to completion for any of the following reasons:

- Voluntary withdrawal of consent

All information contained in this document is confidential and is the sole property of Noctrix Health, Inc.
Any reproduction in part or whole without the written permission of Noctrix Health, Inc. is prohibited.

- Adverse event preventing further study participation
- Investigator believes risk of further subject participation outweighs benefit
- Persistent non-compliance or lost to follow-up
- Pregnancy
- Subject no longer meets inclusion criteria
- Subject makes a significant change to dosage of medications known to interfere with sleep or RLS symptoms, including sleep medications, antidepressants, sedative antihistamines.

The Investigator or research staff will complete a study exit form in the eCRF for any subject who prematurely discontinues from the study. If discontinuation was the result of an AE, the AE will also be recorded in the eCRF.

Upon discontinuation, subjects will receive partial compensation commensurate with their completed Phases.

16.17. Study Termination

The Sponsor may terminate the study as a whole or at individual study sites under the following circumstances:

- Suspicion of risk to subjects, including occurrence of high rate of known AEs or unexpectedly high rate of unexpected AEs
- Poor site compliance with the study protocol
- Inadequate site enrollment
- Obtaining new scientific knowledge that shows that the study is no longer valid or necessary
- Persistent non-compliance with IRB or regulatory requirements
- Persistent failure to comply with obligations arising from the clinical trial agreement
- Other business reasons (e.g., insolvencies or business entity liquidation)

The sponsor will document reasons for study suspension and notify relevant site Investigators and governing IRBs. If suspension occurred because of a safety issue, all Investigators will be notified. When terminating the study, the sponsor and Investigator will assure that adequate consideration is given to the protection of the subjects' interests.

CONFIDENTIAL
All information contained in this document is confidential and is the sole property of Noctrix Health, Inc.
Any reproduction in part or whole without the written permission of Noctrix Health, Inc. is prohibited.

17. Study Endpoints

17.1. Efficacy

17.1.1. Primary Efficacy Endpoint

The primary outcome measure will be responder rate on the CGI-I scale at Week 4 of Phase 1 relative to Baseline (study entry) and compared between the study arms.

Responder rate for the 7-point CGI-I scale will be defined as the proportion of responses of "Much Improved" or "Very Much Improved".

17.1.2. Key Secondary Efficacy Endpoints

For all subjects:

1. PGI-I responder rate (defined as for CGI-I) at Week 4 of Phase 1 relative to Baseline and compared between study arms.
2. Mean reduction in IRLS score at Week 4 of Phase 1, relative to Baseline and compared between study arms.
3. Mean reduction in MOS-II (Medical Outcomes Study Sleep Problems Index I) score at Week 4 of Phase 1, relative to Baseline and compared between study arms.
4. Mean reduction in MOS-II (Medical Outcomes Study Sleep Problems Index II) score at Week 4 of Phase 1, relative to Baseline and compared between study arms.
5. Mean CGI-I score at Week 4 of Phase 1 relative to Baseline and compared between study arms.

Definition and instructions for calculation of the Medical Outcomes Study Sleep Problems Index I and II are described in [13].

17.1.3. Efficacy Analysis

A Statistical Analysis Plan (SAP) will be developed prior to breaking the blind, which will include a plan for efficacy analysis. Briefly, the Intent to Treat population may be used as the primary analysis population for all efficacy analyses, the Per Protocol population may be used as supportive, and missing data may be addressed with the use of multiple imputation. Appropriate statistical techniques will be employed to test statistical significance. If there is success on the primary endpoint, then secondary endpoints may be tested in order, according to the fixed-sequence method, until failure.

CONFIDENTIAL
All information contained in this document is confidential and is the sole property of Noctrix Health, Inc.
Any reproduction in part or whole without the written permission of Noctrix Health, Inc. is prohibited.

The analysis cohort for the primary efficacy endpoint are subjects who are randomized, undergo the assigned treatment, and have complete, evaluable Day 28 data. Subjects will be excluded from the Per Protocol analysis if one or more of the following occurs during or before the Phase of the study corresponding to the endpoint:

1. Dropout or removal from the study.
2. Incomplete or missing data for the endpoint.
3. Clinically significant change in dose or schedule of medication that affects RLS symptoms (IC #8) or major lifestyle changes that affect RLS symptoms to a clinically significant degree (IC #9).
4. New medical information available after randomization indicates an exclusion criterion.
5. Other protocol deviation occurs that is deemed clinically significant by the investigator.

Subjects will also be excluded from the Per Protocol analysis if one or more of the following occurs during the Phase of the study corresponding to the endpoint:

6. Device usage at or after bedtime on fewer than two-thirds of the nights with RLS symptoms at or after bedtime during the Phase, as reported by the subject on the Daily Questionnaire assessments.
7. Missing* two or more follow-ups during the Phase (*Follow-ups completed out-of-window are not considered "missing")
8. Zero total device uses during the Phase.

17.2. Sample Size Justification

Sample size estimates are based on data from use of an earlier prototype of this investigational device in a previously approved protocol (RLS-SNS01). Based on these data and assuming a 1:1 allocation, statistical power of 85%, and 2-sided alpha of 0.05 (or 1-sided alpha of 0.025):

- For CGI-I responder rate, the sample size needed would be 42 subjects, assuming statistical treatment with a two-proportion z-test.
- For PGI-I responder rate, the sample size needed would be 42 subjects, assuming statistical treatment with a two-proportion z-test.

CONFIDENTIAL
All information contained in this document is confidential and is the sole property of Noctrix Health, Inc.
Any reproduction in part or whole without the written permission of Noctrix Health, Inc. is prohibited.

- For mean reduction in IRLS score, the sample size needed would be 112 subjects, assuming statistical treatment with a two-sample t-test.
- For mean reduction in CGI-I score, the sample size needed would be 36 subjects, assuming statistical treatment with a two-sample t-test.

The maximum of these is 112 subjects. Previous data with this investigational device are not available for the MOS-I or MOS-II. Based on estimates of a 15% dropout rate, 132 subjects will be enrolled in the study to yield approximately 112 completed subjects.

17.3. Safety

17.3.1. Safety Endpoints

The safety endpoint will be a descriptive analysis of adverse events (AEs) for both study arms, classified and tabulated by seriousness, relationship to the device, and severity.

17.3.2. Safety Analysis

Frequency charts of adverse events occurring at any time during the study will be produced. The proportion of subjects having one or more adverse events between randomization and the Day 28 will be calculated and compared using a comparison of proportions test. The proportion of subjects reporting on or more adverse events at the Day 56 will tabulated. Similar analyses will be done for SAEs and device-related SAEs. Safety analysis will include all subjects who were treated. A Statistical Analysis Plan (SAP) will be developed prior to breaking the blind, which may include additional plans for safety analysis.

17.4. Blinding Analysis

A Statistical Analysis Plan (SAP) will be finalized prior to breaking the blind, which will include a plan for blinding analysis. The extent to which subjects remained blinded at the end of Phase 1 will be assessed by calculating a blinding index based on data from the Blinding Assessment following Phase 1, in which subjects will be asked what treatment they believe they received. Further, participants who guess that they received “Treatment” or “Sham” (as opposed to “Don’t Know”) will be asked for the primary reason for their guess and these results will be analyzed.

17.5. Exploratory Analysis

Additional statistical analysis that is exploratory in nature may be performed.

CONFIDENTIAL

All information contained in this document is confidential and is the sole property of Noctrix Health, Inc.
Any reproduction in part or whole without the written permission of Noctrix Health, Inc. is prohibited.

18. Remote procedures

During and after the COVID-19 pandemic, replacing some in-person visits with remote interactions may be necessary, for example to reduce risk to subjects and/or to comply with local, state, or federal regulations. In such cases, the study records will indicate which in-person visits were replaced with remote interactions. The allowable study duration may be expanded as needed to allow delay intervals for mailing and receiving programmed devices.

In such cases, one of more of the following approaches may be taken:

1. Remote calibration and training. Remote calibration and training, where possible, will be coordinated by mailing a calibration/training device to the subject, conducting calibration and training via a video call using the calibration/training device, and then mailing programmed devices to the subject using the settings determined during the calibration. Alternatively, the same devices used in the study may be used for calibration/training.
2. Remote follow-up visits. Remote follow-up visits, where possible, will be completed via call or video call, during which the required study assessments will be administered.
3. Devices may be returned using a pre-paid mailing label, as needed.
4. Remote consent. Remote consent procedures will follow the recommendations set forth in the "FDA Guidance on Conduct of Clinical Trials of Medical Products during COVID-19 Public Health Emergency" [14]. For example, or more of the following procedures listed in the guidance will be used to confirm consent:
    a. eConsent via a compliant digital platform;
    b. confirmation of consent by an impartial witness via three-way call or video conference;
    c. remote explanation of consent followed by in-person signature of informed consent document (to minimize duration of in-person interaction).

19. References

CONFIDENTIAL
All information contained in this document is confidential and is the sole property of Noctrix Health, Inc.
Any reproduction in part or whole without the written permission of Noctrix Health, Inc. is prohibited.

1. Allen, R.P. et al. Restless legs syndrome: diagnostic criteria, special considerations, and epidemiology. A report from the restless legs syndrome diagnosis and epidemiology workshop at the National Institutes of Health. Sleep Med. (2003). doi: 10.1016/s1389-9457(03)00010-8
2. 2012 Revised IRLSSG Diagnostic Criteria for RLS. Available at: http://irlssg.org/diagnostic-criteria
3. Allen, R. P. *et al.* Restless legs syndrome prevalence and impact: REST general population study. *Arch. Intern. Med.* (2005). doi:10.1001/archinte.165.11.1286
4. Silber, M.H. Treatment of restless legs syndrome and periodic limb movement disorder in adults. In: UpToDate, Post, TW (Ed), UpToDate, Waltham, MA, 2020
5. García-Borreguero, D. & Williams, A. M. Dopaminergic augmentation of restless legs syndrome. *Sleep Medicine Reviews* (2010). doi:10.1016/j.smrv.2009.11.006
6. Lipford, M.C. and Silber, M.H. Long-term use of pramipexole in the management of restless legs syndrome. Sleep Med. 2012; 13: 1280–1285
7. Hening, W.A. et al. An update on the dopaminergic treatment of restless legs syndrome and periodic limb movement disorder. Sleep (2004). doi: 10.1093/sleep/27.3.560
8. Cornelius, J.R, Tippmann-Peikert, M., Slocumb, N.L., Frerichs, C.F., Silber, M.H. Impulse control disorders with the use of dopaminergic agents in restless legs syndrome: a case-control study. Sleep. 2010 Jan;33(1):81-7.
9. FDA Drug Safety Communication 12-19-2019. Available at: https://www.fda.gov/drugs/drug-safety-and-availability/fda-warns-about-serious-breathing-problems-seizure-and-nerve-pain-medicines-gabapentin-neurontin.
10. Silber, M. H. *et al.* The Appropriate Use of Opioids in the Treatment of Refractory Restless Legs Syndrome. *Mayo Clin. Proc.* (2018). doi:10.1016/j.mayocp.2017.11.007
11. FDA Center for Devices and Radiological Health Guidance Jan 2006. Information sheet guidance for IRBs, clinical investigators, and sponsors: Significant Risk and Nonsignificant risk medical device studies. Available at:

CONFIDENTIAL
All information contained in this document is confidential and is the sole property of Noctrix Health, Inc.
Any reproduction in part or whole without the written permission of Noctrix Health, Inc. is prohibited.

http://www.fda.gov/downloads/RegulatoryInformation/Guidances/UCM126418.pdf

12. Walters AS, LeBrocq C, Dhar A, et al. Validation of the International Restless Legs Syndrome Study Group rating scale for restless legs syndrome. *Sleep Med*. 2003;4(2):121-132. doi:10.1016/s1389-9457(02)00258-7

13. Hays, R. D., & Stewart, A. L. (1992). Sleep measures. In A. L. Stewart & J. E. Ware (eds.), Measuring functioning and well-being: The Medical Outcomes Study approach (pp. 235-259), Durham, NC: Duke University Press.

14. FDA Guidance on Conduct of Clinical Trials of Medical Products during COVID-19 Public Health Emergency. Available at: https://www.fda.gov/media/136238/download

CONFIDENTIAL

All information contained in this document is confidential and is the sole property of Noctrix Health, Inc.
Any reproduction in part or whole without the written permission of Noctrix Health, Inc. is prohibited.
