# Supplementary material for: Efficacy and safety of tonic motor activation (TOMAC) for medication-refractory restless legs syndrome: a randomized clinical trial
Source: Sleep. 2023 Jul 17;46(10):zsad190. doi: 10.1093/sleep/zsad190 (PMC10566236; doi:10.1093/sleep/zsad190)
Supplement: zsad190_suppl_Supplementary_Materials_S2 [file zsad190_suppl_supplementary_materials_s2.pdf]

Efficacy and Safety of Tonic Motor Activation (TOMAC) for Medication-refractory Restless Legs

Syndrome: A Randomized Clinical Trial

Authors:
Richard K. Bogan[1]
Asim Roy[2]
Jerrold Kram[3]
Joseph Ojile[4]
Russell Rosenberg[5]
J. Douglas Hudson[6]
H. Samuel Scheuller[7]
John W. Winkelman[8]
Jonathan D. Charlesworth[9]

Affiliations:
[1]Bogan Sleep Consultants, LLC, Columbia, SC, USA
[2]Ohio Sleep Medicine Institute, Dublin, OH, USA
[3]California Center for Sleep Disorders, San Leandro, CA, USA
[4]Clayton Sleep Institute, LLC, St. Louis, MO, USA
[5]NeuroTrials Research Inc., Atlanta, GA, USA
[6]FutureSearch Trials of Neurology, Austin, TX, USA
[7]Delta Waves, Inc., Colorado Springs, CO, USA
[8]Massachusetts General Hospital, Boston, MA, USA
[9]Noctrix Health, Inc., Pleasanton, CA, USA

Corresponding Author:
Dr. Jonathan D. Charlesworth
Department of Clinical Research
Noctrix Health, Inc.
6700 Koll Center Pkwy, Ste 310
Pleasanton, CA 94566
Phone: (804) 683-4279
jcharlesworth@noctrixhealth.com

DocuSign Envelope ID: 6674474F-5871-479D-AC04-B1AF85994648

STATISTICAL ANALYSIS PLAN

Protocol Number: CT-04

A Multi-Center, Randomized, Double-Blind, Sham-Controlled Study to Evaluate the NTX100 Neuromodulation System for Patients with Medication-Refractory Primary Restless Legs Syndrome (RLS) – The RESTFUL study

April 19, 2022

Prepared for
Noctrix Health, Inc.
6700 Koll Center Parkway, Suite 310
Pleasanton, CA 94566

Prepared by
Roger B. Johnson, Ph.D.
Director, Biostatistics

Reviewed by
R. Leavitt Morrison, M.Sc.
Manager, Biostatistics

Biostatistical Consulting Inc.
91 Hartwell Avenue
Lexington, MA 02421

DocuSign Envelope ID: 6674474F-5871-479D-AC04-B1AF85994648

Signature Page for Analysis Plan

Sponsor: Noctrix Health, Inc.

Study Number: CT-04

Protocol Title: A Multi-Center, Randomized, Double-Blind, Sham-Controlled Study to Evaluate the NTX100 Neuromodulation System for Patients with Medication-Refractory Primary Restless Legs Syndrome (RLS) – The RESTFUL study

Prepared by:<br>Roger B. Johnson, Ph.D.<br>Director of Biostatistics	DocuSigned by:<br>Roger Johnson<br>4A6038A7752042D...<br>Signature	4/20/2022<br>Date
Reviewed by:<br>R. Leavitt Morrison, M.Sc.<br>Manager, Biostatistics	DocuSigned by:<br>Leavitt Morrison<br>6E998BEAA9D0442...<br>Signature	4/19/2022<br>Date

Biostatistical Consulting Inc.
91 Hartwell Avenue
Lexington, MA, 02421

Sponsor:

Jonathan Charlesworth, Ph.D.<br>Vice President, Clinical Research	[signature]<br>Signature	**4/19/2022**<br>Date

Noctrix Health, Inc.
6700 Koll Center Parkway, Suite 310
Pleasanton, CA 94566

Table of Contents

Signature Page for Analysis Plan 2
Table of Contents 3
List of Tables 5
List of Listings 6
List of Figures 7
List of Abbreviations 8
1.0 INTRODUCTION 9
2.0 STUDY OBJECTIVE 11
3.0 STUDY DESIGN 12
3.1 Overview 12
3.2 Method of Assigning Subjects to Treatment 14
3.3 Blinding 14
3.4 Determination of Sample Size 14
3.5 Changes to the Protocol-Specified Analyses 15
4.0 EFFICACY ENDPOINTS 16
4.1 Primary Efficacy Endpoint 16
4.2 Key Secondary Efficacy Endpoints 16
5.0 STATISTICAL CONSIDERATIONS 17
5.1 General Methodology 17
5.2 Adjustments for Covariates 17
5.3 Handling of Dropouts and Missing Data 17
5.4 Interim Analyses 17
5.5 Multicenter Study 18
5.6 Multiple Comparisons / Multiplicity 18
5.7 Examination of Subgroups 18
6.0 ANALYSIS POPULATIONS 19
6.1 Intent-to-Treat (ITT) Population 19
6.2 Per Protocol (PP) Population 19
6.3 Safety Analysis Population 19
7.0 DEMOGRAPHICS AND BASELINE CHARACTERISTICS 20
8.0 EFFICACY AND SAFETY ANALYSES 21
8.1 Primary Efficacy Endpoint Analysis 21
8.2 Key Secondary Efficacy Endpoints Analyses 21
8.3 Safety Analyses 22
9.0 OTHER ANALYSES 24

DocuSign Envelope ID: 6674474F-5871-479D-AC04-B1AF85994648

10.0 REFERENCES 25

Appendix A: TABLE SHELLS
Appendix B: LISTING SHELLS
Appendix C: FIGURE SHELLS

DocuSign Envelope ID: 6674474F-5871-479D-AC04-B1AF85994648

List of Tables

Number	Title
1	Demographics and Baseline Characteristics (ITT Population)
2.1	Primary Efficacy Endpoint (ITT Population)
2.2	Primary Efficacy Endpoint (PP Population)
2.3	Key Secondary Efficacy Endpoints (ITT Population)
2.4	Key Secondary Efficacy Endpoints (PP Population)
2.5	Additional Descriptive Statistics – NTX100 (ITT Population)
2.6	Additional Descriptive Statistics – NTX100 (Per Protocol Population)
2.7	Additional Descriptive Statistics – Sham (ITT Population)
2.8	Additional Descriptive Statistics – Sham (Per Protocol Population)
3.1	Adverse Events (AEs) Overview (Safety Analysis Population)
3.2	Adverse Events (AEs): Incidence by System Organ Class and Preferred Term, and by Treatment and Time Period (Subject Level) (Safety Analysis Population)
3.3	Adverse Events (AEs) by System Organ Class/Preferred Term and Seriousness, and by Treatment and Time Period (Event Level) (Safety Analysis Population)
3.4	Adverse Events (AEs) by System Organ Class/Preferred Term and Severity, and by Treatment and Time Period (Event Level) (Safety Analysis Population)
3.5	Adverse Events (AEs) by System Organ Class/Preferred Term and Relationship to the Device, and by Treatment and Time Period (Event Level) (Safety Analysis Population)
3.6	Adverse Events (AEs): Incidence by Anticipated Observation (AO) Code, and by Treatment and Time Period (Event Level) (Safety Analysis Population)
4	Blinding Analysis (ITT Population)

DocuSign Envelope ID: 6674474F-5871-479D-AC04-B1AF85994648

List of Listings

Number	Title
1.1	Analysis Populations (Randomized Subjects)
1.2	Demographics (ITT Population)
1.3	Refractory Categorization Worksheet (ITT Population)
2.1	Clinical Global Impressions – Improvement (CGI-I) (ITT Population)
2.2	Patient Global Impressions – Improvement (PGI-I) (ITT Population)
2.3	International Restless Legs Syndrome (IRLS) (ITT Population)
2.4	Medical Outcomes Study Sleep Problems (MOS-SLEEP) (ITT Population)
3	Prior and Concomitant Medications (ITT Population)
4	Adverse Events (AEs) (Safety Analysis Population)
5	Blinding Analysis (ITT Population)

List of Figures

Number	Title
1.1	Adverse Events (AEs) Between Randomization and Day 28 by System Organ Class (Safety Analysis Population)
1.2	Adverse Events (AEs) Between Day 28 and Day 56 by System Organ Class (Safety Analysis Population)
2.1	Adverse Events (AEs) Between Randomization and Day 28 by Preferred Term (Safety Analysis Population)
2.2	Adverse Events (AEs) Between Day 28 and Day 56 by Preferred Term (Safety Analysis Population)

List of Abbreviations

**Abbreviation**	**Definition**
AE	Adverse Event
AO	Anticipated Observation
CGI-I	Clinical Global Impressions – Improvement
FDA	Food and Drug Administration
FCS	Fully Conditional Specification
IRLS	International Restless Legs Syndrome Study Group Rating Scale
IRLSS	International Restless Legs Syndrome Society
ITT	Intent-to-Treat
MAR	Missing at Random
MedDRA	Medical dictionary for Regulatory Activities
MI	Multiple Imputation
MOS	Medical Outcomes Study
NPNS	Non-invasive peripheral nerve stimulation
PGI-I	Patient Global Impressions – Improvement
PP	Per Protocol
PT	Preferred Term
RLS	Restless Legs Syndrome
SAE	Serious Adverse Event
SOC	System Organ Class

DocuSign Envelope ID: 6674474F-5871-479D-AC04-B1AF85994648

1.0 INTRODUCTION

This document details the analysis plan for the study entitled "A Multi-Center, Randomized, Double-Blind, Sham-Controlled Study to Evaluate the NTX100 Neuromodulation System for Patients with Medication-Refractory Primary Restless Legs Syndrome (RLS) – The RESTFUL study". It describes the proposed efficacy and safety analyses, including planned summary tables, by-subject data listings, and figures.

A clinical need has been identified of improved treatment for those suffering with primary idiopathic restless legs syndrome (RLS). Patients with RLS have a strong urge with sensations of tingling/pain, usually in their legs, and often present with a primary complaint of not being able to fall asleep regularly. This leads to significant quality of life degradation, depression, daytime sleepiness, lack of productivity, and a host of downstream effects associated with lack of quality sleep.

Restless legs syndrome is a sensorimotor disorder that is characterized by a distressing urge to move the legs and, in some cases, other parts of the body such as arms[1]. The diagnosis is made by a response to five hallmark identifying criteria instituted by the International Restless Legs Syndrome Society (IRLSS)[2], as quoted below:

> "1. An urge to move the legs usually but not always accompanied by or felt to be caused by uncomfortable and unpleasant sensations in the legs.
>
> 2. The urge to move the legs and any accompanying unpleasant sensations begin or worsen during periods of rest or inactivity such as lying down or sitting.
>
> 3. The urge to move the legs and any accompanying unpleasant sensations are partially or totally relieved by movement, such as walking or stretching, at least as long as the activity continues.
>
> 4. The urge to move the legs and any accompanying unpleasant sensations during rest or inactivity only occur or are worse in the evening or night than during the day.
>
> 5. The occurrence of the above features are not solely accounted for as symptoms primary to another medical or a behavioral condition (e.g., myalgia, venous stasis, leg edema, arthritis, leg cramps, positional discomfort, habitual foot tapping)."

Diagnostically, RLS is considered either primary, often occurring within families, or secondary, developing in association with other conditions (such as iron deficiency anemia, pregnancy, or end-stage renal disease).

In the United States, RLS is believed to affect more than 10 million adults and an estimated 1.5 million children and adolescents[3]. About one-third of those with RLS symptoms are bothered sufficiently enough to seek medical attention. Epidemiologic studies also show that women are at least 50% more susceptible to RLS than men and RLS is more common in older adults, although it can occur in some as early as the pre-school years.

The current standard of care involves initial prescription of dopaminergic medications – such as Requip, Mirapex, and Neupro – that initially provide symptomatic relief but often become

DocuSign Envelope ID: 6674474F-5871-479D-AC04-B1AF85994648

ineffective over continued usage[4]. Tolerance to these medications is rapid and well-documented[5]; approximately 10% of patients per year become refractory to these medications, and fewer than 20% patients have sustained benefits lasting 10 years or longer[6]. It is also now understood that dopaminergic medications cause what is known as "augmentation", or paradoxical progressive worsening of RLS symptoms that is much faster than the natural progression of the condition. Due to augmentation, patients on dopaminergic medications require increasingly higher doses[7]. Maximal dosage is limited by an increasing risk of side-effects at higher doses, which include compulsive behaviors including substance abuse, hypersexuality, and gambling[8]. As a result of these downsides of dopaminergic agents, a minority of clinicians are starting to prescribe gabapentinioids (e.g., Horizant) as an alternative first-line of treatment; these medications do not typically lead to augmentation but confer risks such as respiratory depression[9], dizziness, and somnolence during the day.

For the large subpopulation of patients who become refractory to dopaminergic medications – typically due to augmentation – there are no FDA approved treatment options and no safe treatment options. As a result of tolerance, augmentation, and dosage limitations, RLS patients often continue to suffer from moderate-severe RLS symptoms while continuing to be reliant on high doses of dopaminergic medications to provide a small degree of relief. To address the massive unmet need, the leading clinicians involved with RLS advocate prescribing off-label opioids[10]. The leading options, oxycodone and methadone, have well documented risks, which include addiction, dependence, overdose, and occasionally death. This situation is especially concerning because primary RLS typically starts in middle age or earlier and persists throughout life, thus patients may end up reliant on opioids for the final decades of their lives.

The investigational device – the NTX100 Neuromodulation System – is a non-invasive nerve peripheral stimulation (NPNS) device developed by Noctrix Health, Inc. (Sponsor) and is designed to bilaterally stimulate the common peroneal nerve. Stimulation electrodes are positioned superficially and bilaterally on the lower legs over the head of the fibula bone, a position where the peroneal nerve is closest to the skin. This nerve target innervates regions of the lower extremities commonly associated with RLS symptoms.

This study evaluates the effects of NPNS on the symptoms of RLS during in-home subject-administered stimulation. This approach is useful for evaluating safety, usability, tolerability, and preliminary efficacy in a realistic environment, thus identifying any and all barriers to effective and tolerable use.

DocuSign Envelope ID: 6674474F-5871-479D-AC04-B1AF85994648

2.0 STUDY OBJECTIVE

The study objective is to provide comparative evidence assessing clinically meaningful benefit in the treatment of patients with moderate to severe medication-refractory RLS with the NTX100 Neuromodulation System.

DocuSign Envelope ID: 6674474F-5871-479D-AC04-B1AF85994648

3.0 STUDY DESIGN

3.1 Overview

The study consists of a 4-week prospective, randomized, sham-controlled, double-blinded phase (Phase 1) followed by a 4-week prospective open-label phase (Phase 2) for a total of 8-weeks of follow-up per subject. The design of the trial is illustrated in the flowchart below (Figure 1).

Phase 1 (4 weeks): Subjects who pass screening will be randomly allocated at a 1:1 ratio to receive Active or Sham treatment throughout the 4-week duration of Phase 1. The primary endpoint and key secondary efficacy endpoints #1-5 will compare the final week of Phase 1 to study entry ("Baseline").

Phase 2 (4 weeks): All subjects who complete Phase 1 will be allocated to receive open-label Active treatment throughout the 4-week duration of Phase 2. Key secondary efficacy endpoint #6 will compare the final week of Phase 2 to Baseline among subjects who receive Active treatment during both Phase 1 and Phase 2.

Figure 1: Study Design Flowchart

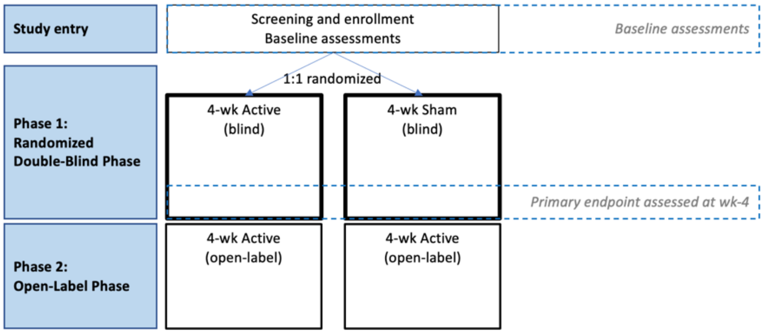

The schedule of assessments for this study is presented in Table 1.

DocuSign Envelope ID: 6674474F-5871-479D-AC04-B1AF85994648

Table 1: Schedule of Assessments

	**EVAL 1**	EVAL 2	EVAL 3	**EVAL 4**	EVAL 5	**EVAL 6**	EVAL 7	EVAL 8	EVAL 9	EVAL 10	**EVAL 11**
	Day 0* *in-office*	Day 3	Day 7	Day 14 *in-office*	Day 21	Day 28 *in-office*	Day 31	Day 35	Day 42	Day 49	Day 56 *in-office*
Informed Consent	X										
Screening	X										
Subject characterization	X										
Medical History	X										
Refractory categorization	X										
Concomitant Medications	X			X		X			X		X
Randomized receipt of Treatment or Sham Device	X										
Begin open-label use of Treatment Device						X					
Daily Questionnaire follow-up		X	X	X	X	X	X	X	X	X	X
Weekly questionnaire and follow-up			X	X	X	X		X	X	X	X
CGI-I						X					X
PGI-I				X		X			X		X
IRLS	X			X		X			X		X
MOS-Sleep	X					X					X
Custom RLS questionnaire						X					X
Blinding Assessment						X					
Adverse Events		X	X	X	X	X	X	X	X	X	X

** If EVAL 1 spans multiple days, Day 0 refers to the final day of EVAL 1.*

3.2 Method of Assigning Subjects to Treatment

Subjects who meet eligibility criteria will be randomized in a 1:1 ratio for Phase 1 to either:

- NTX100 programmed to ACTIVE mode
- NTX100 programmed to SHAM mode

Randomization assignments will be stratified by study center with randomly chosen block sizes of 4 or 6.

3.3 Blinding

Phase 1 will be double-blind. Information about treatment assignment will be concealed from subjects, investigators, and site staff, except as required in cases of safety emergencies. In additional, all sponsor staff interacting directly with the research staff or investigators (e.g., in a monitoring capacity) or subjects (e.g., in a device specialist capacity) will be blinded to the Phase 1 treatment assignment throughout the study (even during Phase 2).

Phase 2 will be open-label. At the beginning of Phase 2, the treatment assignment on each device will be set to Active for all subjects. This will take place without revealing the previous treatment assignment during Phase 1.

Active and sham devices will be physically identical and will provide identical visual feedback during operation. Both active and sham mode will have an initial ramp-up in stimulation intensity lasting approximately 30 seconds and during which sensations are typically noticeable. No subjects with prior experience with NTX100 nor prior experience using any neurostimulation devices to treat RLS symptoms will be enrolled.

The investigator must contact the sponsor if the need to unblind arises. The Investigator should not unblind UNLESS knowledge of the subject's treatment assignment is required for the subject's clinical care and safety. Documentation of breaking the blind should be recorded in the subject's *Narrative eCRF* with the date and time the blind was broken, and the names of the personnel involved.

3.4 Determination of Sample Size

Sample size estimates were based on data from use of an earlier prototype of this investigational device in a previously approved protocol (RLS-SNS01). Based on these data and assuming a 1:1 allocation to treatment, statistical power of 85%, and a two-sided alpha of 0.05 (or one-sided alpha of 0.025):

- For CGI-I responder rate at Week 4 of Phase 1, the sample size needed is 42 subjects, assuming statistical analysis with a two-proportion normal approximation test.
- For PGI-I responder rate at Week 4 of Phase 1, the sample size needed is 42 subjects, assuming statistical analysis with a two-proportion normal approximation test.
- For reduction in IRLS score at Week 4 of Phase 1, the sample size is 112 subjects, assuming statistical analysis with a two-sample t-test.
- For CGI-I score at Week 4 of Phase 1 relative to Baseline, the sample size needed is 36 subjects, assuming statistical analysis with a two-sample t-test.

DocuSign Envelope ID: 6674474F-5871-479D-AC04-B1AF85994648

The maximum of these sample sizes is 112 subjects. Previous data with this investigational device are not available for the MOS-I or MOS-II. Based on estimates of a 15% dropout rate, approximately 132 subjects will be enrolled in the study to yield approximately 112 completed subjects.

3.5 Changes to the Protocol-Specified Analyses

Reduction in IRLS Question #7 score (“How often do you get RLS symptoms?”) from Baseline to Week 8 was added as a key secondary efficacy endpoint for subjects assigned to NTX100 in both phases of the study. A summary of the same reduction from Week 4 to Week 8 was added for subjects switching from Sham to NTX100 after Week 4.

4.0 EFFICACY ENDPOINTS

4.1 Primary Efficacy Endpoint

The primary efficacy endpoint is the response on the Clinical Global Impressions-Improvement (CGI-I) scale at Week 4 of Phase 1 relative to Baseline (study entry). A "successful" response for the 7-point CGI-I scale will be defined as a response of "Much Improved" or "Very Much Improved".

4.2 Key Secondary Efficacy Endpoints

The key secondary efficacy endpoints are as follows:

1. PGI-I response (defined as for CGI-I) at Week 4 of Phase 1 relative to baseline.
2. Reduction in IRLS score at Week 4 of Phase 1 relative to Baseline.
3. Reduction in MOS-II (Medical Outcomes Study Sleep Problems Index I) score at Week 4 of Phase 1 relative to Baseline.
4. Reduction in MOS-I (Medical Outcomes Study Sleep Problems Index II) score at Week 4 of Phase 1, relative to Baseline and compared between study arms.
5. CGI-I score at Week 4 of Phase 1 relative to Baseline.
6. For subjects assigned to NTX100 in both Phases 1 and 2, reduction in IRLS Question #7 score ("How often do you get RLS symptoms?") from Baseline to Week 8.

DocuSign Envelope ID: 6674474F-5871-479D-AC04-B1AF85994648

5.0 STATISTICAL CONSIDERATIONS

5.1 General Methodology

The statistical analysis of the data obtained from this study will be performed using SAS® version 9.4 or higher.

Data collected in this study will be documented using summary tables, subject data listings, and figures. Continuous variables will be summarized using descriptive statistics, specifically the mean, median, standard deviation, minimum and maximum. Categorical variables will be summarized using frequencies and percentages. For continuous data, the minimum and the maximum will use the same decimal place accuracy as the raw data. The mean, median, and standard deviation will use one more decimal place than the raw data. For categorical data, percentages will be reported to one decimal place. P-values will be reported to 4 decimal places. P-values less than 0.0001 will be displayed as <0.0001 in the tables. All statistical tests for efficacy will be performed at the one-sided 0.025 significance level, and all statistical tests for safety (adverse events) will be performed at the two-sided 0.05 significance level.

Data listings will be sorted by site number and subject ID number.

5.2 Adjustments for Covariates

There will be no adjustment for covariates in any efficacy or safety analysis, unless there are missing data for the primary efficacy endpoint and multiple imputation methods are used (see Section 5.3). Only the logistic regression analysis conducted to assess the blinding will include adjustment for covariates. The dependent variable for this analysis will be the participant's guessed treatment, and the explanatory variables will be the participant's actual treatment and the following two PGI-I Day 28 indicator variables as covariates: (1) being at least "minimally improved" and (2) being at least "much improved".

5.3 Handling of Dropouts and Missing Data

If there are any missing data for the primary efficacy endpoint, multiple imputation (MI) methods will be used to impute the missing data. Using SAS® PROC MI, the logistic regression method of imputation will be used with treatment group and the following covariates as explanatory variables: IRLS at Days 0 and 14 and PGI-I at Day 14. This method of multiple imputation is appropriate for a binary dependent variable with explanatory variables following a monotone missing pattern and assumes that the data for the dependent variable are missing at random (MAR). If the data do not follow a monotone missing pattern, the fully conditional specification (FCS) method will be used to impute missing data. For each imputed dataset, PROC FREQ of SAS® will be used to obtain the responder rate and the corresponding standard error for each treatment. PROC MIANALYZE of SAS® will be used to combine the results from the imputed datasets to produce an overall estimate of the true responder rate and the corresponding 95% confidence interval for each treatment. The estimated difference in true responder rates and the p-values for the test for superiority of NTX100 to sham control from a normal approximation test will also be presented.

5.4 Interim Analyses

No interim analyses are planned.

DocuSign Envelope ID: 6674474F-5871-479D-AC04-B1AF85994648

5.5 Multicenter Study

This is a multicenter study. Four to eight clinical sites within the United States will participate in the study.

5.6 Multiple Comparisons / Multiplicity

The fixed sequence method of statistical testing will be used for the primary and key secondary efficacy endpoints. In order to control the overall type I error rate, the key secondary efficacy endpoints will be tested in a hierarchical, gatekeeping manner in the order specified in Section 4.2, and only if primary efficacy endpoint success has been achieved. If one key secondary efficacy endpoint does not meet statistical significance in favor of NTX100, formal testing of subsequent key secondary efficacy endpoints will not be conducted.

5.7 Examination of Subgroups

No subgroup analyses are planned.

6.0 ANALYSIS POPULATIONS

6.1 Intent-to-Treat (ITT) Population

The Intent-to-Treat (ITT) Population will include all eligible subjects who pass screening and are randomized into the study.* The ITT Population Set will be used for the primary analysis of all efficacy endpoints, and subjects will be analyzed based on the treatment to which they are randomized.

6.2 Per Protocol (PP) Population

The Per Protocol (PP) population will include all subjects who pass screening, are randomized into the study, undergo the assigned treatment, and have complete and evaluable Day 28 data, except that:

(i) Subjects will be excluded from the Per Protocol Population if one or more of the following occurs during or before the Phase of the study corresponding to the endpoint:

1. Dropout or removal from the study.
2. Incomplete or missing data for the endpoint.
3. Clinically significant change in dose or schedule of one of the following classes of medication during the study: RLS medications, antidepressants, sleep medications, or sedative antihistamines.
4. New medical information available after randomization indicates an exclusion criterion.
5. Other protocol deviation occurs that is deemed clinically significant by the investigator.

(ii) Subjects will also be excluded from the Per Protocol Population if one or more of the following occurs during the Phase of the study corresponding to the endpoint:

6. Device usage on fewer than two-thirds of the nights with RLS symptoms as reported by the subject on the Daily Questionnaire assessments.
7. Missing two or more follow-ups during the Phase (Note: Follow-ups completed out-of-window are not considered "missing")
8. Zero total device uses during the Phase.

The PP Population will be used for a secondary analysis of all efficacy endpoints.

6.3 Safety Analysis Population

The Safety Analysis Population will include all subjects who receive any dose of study treatment, including subjects who undergo calibration but fail screening. All analyses of adverse events will be based on this population, and subjects will be analyzed based on the actual treatment received.

*Subject 05-002 failed screening and was randomized in error; however, this error was detected prior to treatment. Because the subject did not pass screening or receive treatment, they will be excluded from the ITT population.

7.0 DEMOGRAPHICS AND BASELINE CHARACTERISTICS

Demographics and baseline characteristics will be summarized for the ITT Population. Descriptive statistics will be presented for the continuous variables of age, height, weight, BMI, IRLS total score at baseline, and the duration of RLS symptoms. Frequencies and percentages will be presented for the categorical variables of sex (at birth), ethnicity, race, category of medication that the subject is refractory to, and categories of medication that the subject is currently taking for the indication of RLS, among the following categories: Dopamine agonist, Alpha-2-delta ligand, Opioid, Benzodiazepine, and Other.

DocuSign Envelope ID: 6674474F-5871-479D-AC04-B1AF85994648

8.0 EFFICACY AND SAFETY ANALYSES

8.1 Primary Efficacy Endpoint Analysis

The primary efficacy endpoint is the response on the Clinical Global Impressions-Improvement (CGI-I) scale at Week 4 of Phase 1 relative to Baseline (study entry) where a "successful" response is defined as a response of "Much Improved" or "Very Much Improved". This endpoint will be summarized by treatment group using frequencies and percentages. The null and alternative hypotheses for this endpoint are as follows:

H_0: $RR_T \leq RR_S$

versus

H_1: $RR_T > RR_S$

where RR_T and RR_S are the true responder rates for the NTX100 treatment group and the sham control group, respectively. The null hypothesis will be tested using a one-sided normal approximation test for the comparison of two proportions at the alpha = 0.025 level. If there are any missing data for this endpoint, multiple imputation (MI) methods will be used to impute the missing data.

8.2 Key Secondary Efficacy Endpoints Analyses

The key secondary efficacy endpoints below will be analyzed using a fixed sequential method in the order they are listed. Secondary efficacy endpoint #1 will only be analyzed if the primary efficacy endpoint analysis results in rejection of the null hypothesis, and subsequent secondary efficacy endpoints will only be analyzed if all prior secondary efficacy endpoint analyses resulted in rejection of the null hypothesis (e.g., secondary efficacy endpoint #3 would only be tested if the primary analyses of the primary and first two secondary efficacy endpoints result in rejection of the null hypothesis). Analyses of subsequent secondary endpoints will continue hierarchically until the first analysis that results in failure to reject the null hypothesis, and some or all of the secondary efficacy endpoint analyses may not be formally performed.

The key secondary efficacy endpoints are as follows:

1. PGI-I response (defined as for CGI-I) at Week 4 of Phase 1 relative to Baseline.
2. Reduction in IRLS score at Week 4 of Phase 1 relative to Baseline.
3. Reduction in MOS-II score at Week 4 of Phase 1 relative to Baseline.
4. Reduction in MOS-I score at Week 4 of Phase 1 relative to Baseline.
5. CGI-I score at Week 4 of Phase 1 relative to Baseline.
6. For subjects assigned to NTX100 in both Phases 1 and 2, reduction in IRLS Question #7 score ("How often do you get RLS symptoms?") from Baseline to Week 8.

PGI-I response at Week 4 of Phase 1 relative to Baseline will be analyzed in the same manner as the primary efficacy endpoint, except that MI methods will not be used if there are missing data.

The reduction in IRLS score at Week 4 of Phase 1 relative to Baseline will be summarized by treatment group using descriptive statistics. The null and alternative hypotheses for this endpoint are as follows:

DocuSign Envelope ID: 6674474F-5871-479D-AC04-B1AF85994648

H_0: $\mu_T \leq \mu_S$
versus
H_1: $\mu_T > \mu_S$

where μ_T and μ_S are the true means for the NTX100 treatment group and the sham control group, respectively. The null hypothesis will be tested using a one-sided, two-sample t-test at the alpha = 0.025 level.

The reductions in MOS-I and MOS-II scores at Week 4 of Phase 1 relative to Baseline will be analyzed in the same manner as the reduction in IRLS score at Week 4 of Phase 1 relative to Baseline.

The CGI-I score at Week 4 of Phase 1 relative to Baseline will be summarized by treatment group using descriptive statistics. The null and alternative hypotheses for this endpoint are as follows:

H_0: $\mu_T \geq \mu_S$
versus
H_1: $\mu_T < \mu_S$

where μ_T and μ_S are the true means for the NTX100 treatment group and the sham control group, respectively. The null hypothesis will be tested using a one-sided, two-sample t-test at the alpha = 0.025 level.

For subjects assigned to NTX100 in both Phases 1 and 2, the reduction in IRLS Question #7 score ("How often do you get RLS symptoms?") from Baseline to Week 8 will be summarized using descriptive statistics. The null and alternative hypotheses for this endpoint are as follows:

H_0: $\mu_{TT} \leq 0$
versus
H_1: $\mu_{TT} > 0$

where μ_{TT} is the true mean reduction for subjects who received NTX100 in both Phases 1 and 2. The null hypothesis will be tested using a one-sided, one-sample t-test at the alpha = 0.025 level.

8.3 Safety Analyses

The proportion of subjects having one or more adverse events (AEs) with new onset or worsening (relative to baseline) between randomization and Day 28 will be presented by actual treatment group. The reporting of AEs will include anticipated observations (AOs), which are specific predefined categories of mild AEs that are potentially associated with the use of non-invasive peripheral nerve stimulation devices such as the study device. A two-sided Fisher's Exact Test will be performed to test the null hypothesis that the true proportions are equal for the two treatments versus the alternative hypothesis that they are not equal. The proportion of subjects reporting new onset or worsening (relative to baseline and/or the period between randomization and Day 28) AEs between Day 28 and Day 56 will be presented for NTX100

DocuSign Envelope ID: 6674474F-5871-479D-AC04-B1AF85994648

treatment. Similar analyses will be done for serious adverse events (SAEs), device-related SAEs, and anticipated observations (AOs).

Adverse events will be coded using the Medical Dictionary for Regulatory Activities (MedDRA). AEs will be summarized at the subject level using counts and percentages by MedDRA system organ class (SOC) and preferred term (PT). AEs will also be summarized at the event level using counts and percentages by SOC/PT and seriousness, by SOC/PT and severity, and by SOC/PT and relationship to the device. AEs will also be summarized at the event level using counts and percentages by Anticipated Observation (AO) code. All AE tables will summarize AEs occurring between randomization and Day 28 separately from AEs occurring between Day 28 and Day 56. AEs reported for each period will be those AEs with new onset or worsening (if present prior to the period) occurring within the period.

Frequency charts of adverse events occurring between randomization and Day 28 and between Day 28 and Day 56 will be produced separately. The proportion of subjects with at least one AE will be presented overall and by SOC. The proportion of subjects with at least one occurrence of individual AEs will be presented for AEs occurring in >3% of subjects overall or within either arm.

9.0 OTHER ANALYSES

For subjects assigned to NTX100 in both Phases 1 and 2, frequencies and percentages or descriptive statistics, as appropriate, will be presented for the following variables:

- CGI-I response at Week 8
- PGI-I response at Week 8
- Reduction in IRLS total score from Baseline (Study entry) to Week 8
- Reduction in MOS-II score from Baseline (Study entry) to Week 8
- Reduction in MOS-I score from Baseline (Study entry) to Week 8
- CGI-I score at Week 8

For subjects assigned to Sham in Phase 1 and NTX100 in Phase 2, frequencies and percentages or descriptive statistics, as appropriate, will be presented for the following variables:

- Change in CGI-I responder rate from Week 4 to Week 8 (Week 8 – Week 4)
- Change in PGI-I responder rate from Week 4 to Week 8 (Week 8 – Week 4)
- Reduction in IRLS total score from Week 4 to Week 8
- Reduction in MOS-II score from Week 4 to Week 8
- Reduction in MOS-I score from Week 4 to Week 8
- Change in CGI-I score from Week 4 to Week 8
- Reduction in IRLS Question #7 score ("How often do you get RLS symptoms?") from Week 4 to Week 8

A blinding assessment will be conducted following Phase 1, in which participants will be asked what treatment they believe they received. The study participants will state their belief regarding their treatment group assignment (i.e., "Treatment", "Sham", or "Don't Know"). A logistic regression analysis will be conducted using data collected at the blinding assessment to assess the blinding. The dependent variable for this analysis will be the participant's guessed treatment, and the explanatory variables will be the participant's actual treatment and the following two PGI-I Day 28 indicator variables: (1) being at least "minimally improved" and (2) being at least "much improved". The PGI-I variables will be used as measures of treatment efficacy. Cases for which the guessed treatment is "Don't Know" or missing will be excluded from the analysis. The estimated coefficient for the actual treatment will be presented, together with the p-value for testing the null hypothesis that the coefficient equals 0 versus the alternative hypothesis that the coefficient does not equal 0. This analysis will be based on the ITT Population. The purpose of the blinding analysis is to evaluate whether there is statistical evidence that the study blinding was compromised. The approach to be taken is to examine whether the actual treatment is predictive of the participant's guessed treatment, after taking into account treatment efficacy.

Further, participants who guess "Treatment" or "Sham" (as opposed to "Don't Know") will be asked for the primary reason for their guess and the results will be summarized by treatment group using frequencies and percentages.

DocuSign Envelope ID: 6674474F-5871-479D-AC04-B1AF85994648

10.0 REFERENCES

1. Allen, R.P., et al. Restless legs syndrome: diagnostic criteria, special considerations, and epidemiology. A report from the restless legs syndrome diagnosis and epidemiology workshop at the National Institutes of Health. *Sleep Med*. (2003). doi: 10.1016/s1389-9457(03)00010-8.
2. 2012 Revised IRLSSG Diagnostic Criteria for RLS. Available at: http://irlssg.org/diagnostic-criteria.
3. Allen, R. P., *et al.* Restless legs syndrome prevalence and impact: REST general population study. *Arch. Intern. Med.* (2005). doi:10.1001/archinte.165.11.1286.
4. Silber, M.H. Treatment of restless legs syndrome and periodic limb movement disorder in adults. In: UpToDate, Post, TW (Ed), UpToDate, Waltham, MA, 2020.
5. García-Borreguero, D. & Williams, A. M. Dopaminergic augmentation of restless legs syndrome. *Sleep Medicine Reviews* (2010). doi:10.1016/j.smrv.2009.11.006.
6. Lipford, M.C. and Silber, M.H. Long-term use of pramipexole in the management of restless legs syndrome. *Sleep Med*. 2012; 13: 1280–1285.
7. Hening, W.A., et al. An update on the dopaminergic treatment of restless legs syndrome and periodic limb movement disorder. *Sleep* (2004). doi: 10.1093/sleep/27.3.560.
8. Cornelius, J.R, Tippmann-Peikert, M., Slocumb, N.L., Frerichs, C.F., Silber, M.H. Impulse control disorders with the use of dopaminergic agents in restless legs syndrome: a case-control study. *Sleep*. 2010 Jan;33(1):81-7.
9. FDA Drug Safety Communication 12-19-2019. Available at: https://www.fda.gov/drugs/drug-safety-and-availability/fda-warns-about-serious-breathing-problems-seizure-and-nerve-pain-medicines-gabapentin-neurontin.
